# DNA methylation fine-tunes pro-and anti-inflammatory signalling pathways in inactive ulcerative colitis tissue biopsies

**DOI:** 10.1038/s41598-024-57440-0

**Published:** 2024-03-21

**Authors:** Wei Meng, Christopher G. Fenton, Kay-Martin Johnsen, Hagar Taman, Jon Florholmen, Ruth H. Paulssen

**Affiliations:** 1 Clinical Bioinformatics Research Group, Department of Clinical Medicine, Faculty of Health Sciences, UiT- The Arctic University of Norway, Tromsø, Norway; 2 Gastroenterology and Nutrition Research Group, Faculty of Health Sciences, UiT- The Arctic University of Norway, Tromsø, Norway; 3 Department of Medical Gastroenterology, University Hospital of North Norway, Tromsø, Norway; 4 Genomics Support Centre Tromsø, Department of Clinical Medicine, Faculty of Health Sciences, UiT- The Arctic University of Norway, Sykehusveien 44, 9037 Tromsø, Norway

**Keywords:** Ulcerative colitis, Remission, DNA methylation, Epigenetics, Computational biology and bioinformatics, Gastrointestinal diseases

## Abstract

DNA methylation has been implied to play a role in the immune dysfunction associated with inflammatory bowel disease (IBD) and the disease development of ulcerative colitis (UC). Changes of the DNA methylation and correlated gene expression in patient samples with inactive UC might reveal possible regulatory features important for further treatment options for UC. Targeted bisulfite sequencing and whole transcriptome sequencing were performed on mucosal biopsies from patients with active UC (UC, n = 14), inactive UC (RM, n = 20), and non-IBD patients which served as controls (NN, n = 11). The differentially methylated regions (DMRs) were identified by DMRseq. Correlation analysis was performed between DMRs and their nearest differentially expressed genes (DEGs). Principal component analysis (PCA) was performed based on correlated DMR regulated genes. DMR regulated genes then were functional annotated. Cell-type deconvolutions were performed based on methylation levels. The comparisons revealed a total of 38 methylation-regulated genes in inactive UC that are potentially regulated by DMRs (correlation p value < 0.1). Several methylation-regulated genes could be identified in inactive UC participating in IL-10 and cytokine signalling pathways such as IL1B and STAT3. DNA methylation events in inactive UC seem to be fine-tuned by the balancing pro- and anti- inflammatory pathways to maintain a prevailed healing process to restore dynamic epithelium homeostasis.

## Introduction

Ulcerative colitis (UC) is a chronic inflammatory disorder of the colon with a relapsing course^1^. To achieve remission, long-term treatment is often required^2^. Multiple factors can cause the disease, and research has focused on investigating genetic susceptibility, microbiome communities, environmental factors, and immune responses in UC patients^3–5^ Current research denotes the importance of interactions between inflammation and environmental factors^6–8^. Therefore, dynamic processes such as DNA methylation, have been implied to play a role in the immune dysfunction associated with inflammatory bowel disease (IBD)^9–11^ and disease development of UC^12–15^. DNA methylation in active UC has been reported recently^9,10,12,13^. Variations in DNA methylation patterns have been associated with homeostasis and defence, immune responses, and progression and development of colorectal cancer (CRC)^12,13^.

However, DNA methylation during UC in remission has not been explored in detail. Long-term treatment of UC patients is often necessary to achieve induction and maintenance of clinical remission^16–18^. Previous work on transcriptional signatures in UC has revealed that UC in remission is a permanently altered state of UC with a still ongoing quiescent inflammation^19,20^. Therefore, induced epigenetic changes due to long-term treatment can be expected. In this study, the genome-wide DNA methylome in a UC remission patient cohort was investigated to see if methylation contributes to the expression regulation of specific molecular signatures recently found in a patient cohort with different remission duration^21^. It is believed that changes in the DNA methylation status in remission patients, when correlated to differentially expressed genes (DEGs), may reveal possible regulatory features important for further treatment options for UC patients.

## Materials and methods

### Patient material

Mucosal biopsies (n = 43) were collected with a standardised sampling method of 12 newly diagnosed, treatment-naïve UC patients with mild to moderate disease activity, 20 patients with inactive UC (remission) and 11 control subjects from a former study^21^. Controls were derived from subjects performing a cancer screening, with normal colonoscopy and normal colonic histological examination. UC was diagnosed based upon established clinical endoscopic and histological criteria as defined by the ECCO guidelines^22^. The grade of inflammation was assessed during colonoscopy using the UC disease activity index (UCDAI) endoscopic sub-score with 3 to 10 for mild to moderate disease^23^. TNF-α mRNA expression levels were measured by real-time PCR to determine disease activity. All UC patients were initially treated with 5-aminosalicylic acid (5-ASA), in some cases also supplemented with immunosuppressive drugs (Imurel, methotrexate (MTX), and Infliximab) until disease remission. Patients with achieved clinical and endoscopic remission and normalised TNF-α levels were included in this study, with a defined UCDAI score ≤ 2, an endoscopic sub-score of 0 or 1, and TNFα-levels < 7500 copies/μg protein. All patient characteristics are depicted in Table 1. The samples were taken from an established Biobank approved by the Norwegian Board of Health (952/2006). The study participants signed informed and written consent forms. Approvals were granted by the Regional Committee of Medical Ethics of Northern Norway, Ref no: 14/2004, 1349/2012 and 29895/2020.

**Table 1:** Patient characteristics.

Characteristics	Controls (n = 11)	UC remission (n = 20)	Active UC§# (n = 14)
Gender (male/female)	8/3	10/10	9/5
Age (years) mean ± SD	52.2 ± 19.3	50.0 ± 13.5	40.7 ± 13.9
Clinical score ± SD	0	0	7.78 ± 1.52
Endo Score mean ± SD	0	0.25 ± 0.50¶	1.79 ± 0.43
Geboes score (total) ± SD	n.d	0.13 ± 0.70¤	6.35 ± 2.93
TNF-α copies/µg RNA ± SD	4366 ± 1998	4500 ± 1509*	15,907 ± 9623
Calprotectin (mg/kg) mean ± SD	n.d	17.0 ± 53.78€	587.5 ± 483.8¥
Extension of disease£	_	2/12/6	2/9/3
Medication#	_	20/0/6	_

*TNF-α copies/µg RNA in 16 patients.

£ proctitis/left-sided colitis/pancolitis.

^#^5-ASA/steroids/immunosuppressives.

¤Average score of 13 patients.

€Average calprotectin levels in 15 patients.

¥Average calprotectin levels in 11 patients.

^¶^Average score of 16 patients.

^§^Data adapted for comparison from Fenton et al.^20^.

± SD, Standard Deviation.

### DNA and RNA isolation

Genomic DNA and total RNA was isolated using the Allprep DNA/ RNA Mini Kit from Qiagen (catalogue number 80204) and the QIAcube instrument (Qiagen), according to the manufacturer's protocol. The quantity and purity of both DNA and RNA were assessed by using the NanoDrop ND-1000 spectrophotometer (Thermo Fisher Scientific, Wilmington, DE). The Experion Automated Electrophoresis System (Bio-Rad, Hercules, CA.) and the RNA StdSens Analysis Kit (Bio-Rad, catalogue number 700–7103) were used to evaluate RNA integrity. All RNA samples used for analyses showed an RNA integrity number (RIN) value between 8.0 and 10.0. DNA and RNA samples were kept at − 70°C until further use.

### Institutional review board statement

The study was conducted according to the guidelines of the Declaration of Helsinki. Approvals were granted by the Regional Committee of Medical Ethics of Northern Norway, Ref no: 14/2004, 1349/2012 and 29,895/2020.

### Informed consent statement

Written informed consent has been obtained from the study participants to publish this paper.

## Library preparation and next-generation sequencing

The libraries were prepared using the SeqCap Epi CpGiant Enrichment Kit (Roche, Switzerland) which enables the targeting of selected genomic regions from bisulfite-treated genomic DNA to identify specific regions in the genome for methylation variation assessment and as previously described^13^. DNA was bisulfite converted using the EZ DNA Methylation-lightning Kit (Zymo Research, USA, cat no: D5030) prior to the hybridisation step and according to the manufacturer’s instructions. The amount of input material was 1060 ng of genomic DNA per sample. DNA library quality was assessed using the Bioanalyzer 2100 and the Agilent DNA 1000 kit (cat no: 5067–1504, Agilent Technologies, Santa Clara, USA), according to the manufacturer's instructions. DNA libraries generated fragments with an average size of 322 bp. DNA libraries were diluted to 4nM before sequencing. Whole transcriptome libraries were prepared with the TruSeq Stranded Total RNA LT Sample Prep Kit from Illumina (cat no: RS-122–2203). The amount of input material was 1μg of total RNA. The Bioanalyzer 2100 and the Agilent DNA 1000 kit (cat no: 5067–1504, Agilent Technologies, Santa Clara, USA) were used to assess the quality of the RNA libraries. RNA libraries generated fragments with an average size of 301 bp. Libraries were normalised to 10 nM and diluted to 4 nM prior to sequencing. DNA and RNA libraries were sequenced on the NextSeq 550 instrument, using a high output flow cell 150 cycles (cat no: FC-404–2002, Illumina, USA) and according to the manufacturer's instruction. The libraries were sequenced using paired-end mode.

### Data analysis

**Figure 1:**
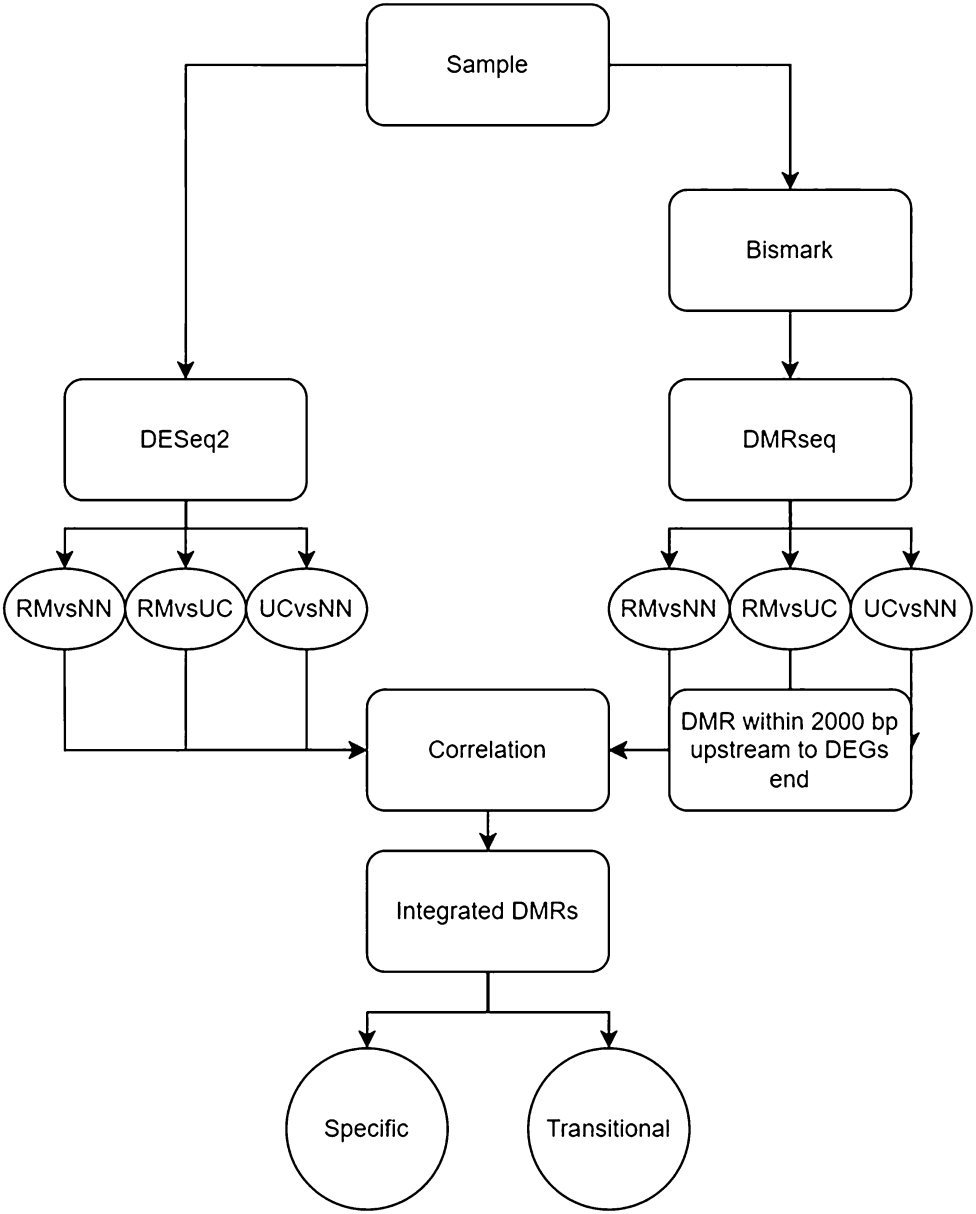
Flow chart for generating differentially methylated region (DMR) correlated gene patterns for groups of inactive UC (RM), active UC (UC), and controls (NN). Processed sequencing data of 45 samples underwent Bismark and DMRseq, incorporating three comparisons to identify DMRs (q value < 0.05) and underwent DEseq2 for DEGs (p.adj < 0.05). DMRs located within 2000 bp upstream of DEGs were correlated with the DEGs. The correlated DMRs were then integrated. Integrated DMRs were grouped based on expression level and t-test on methylation level (p < 0.05) to ensure a fit with the pattern conditions.

A flow chart illustrating the downstream analysis process is shown in Fig. 1.

### RNA sequencing

Quality scoring, base calling, and adapter removal were performed on the Illumina NextSeq 550 sequencing instrument. The output FastQ file was aligned with reference GENCODE Human Release 33 (Human Genome Assembly GRCh38.p13) ((https://www.ncbi.nlm.nih.gov/grc/human/data) by Kallisto^24^. DEseq2 was used to generate a list of differential expressed gene transcripts (DEGs)^25^. DEGs with only p.adj < 0.05 were kept.

### Bisulfite converted DNA and DMRSeq

Quality scoring, base calling, and adapter removal were performed on the Illumina NextSeq 550 sequencing instrument. The output FastQ file was aligned with reference GENCODE Human Release 38 by Bismark with Bowtie2^26,27^. The output BAM files were then used to generate coverage data and methylation data by using Bismark methylation extractor^27^. The results of Bismark were further processed with DMRseq^28^ to find differentially methylated regions. Only differentially methylated regions (DMRs) with a q-value < 0.05 were kept. In this context, the q-value served as an adjustment for multiple comparison to control the false discovery rate.

### Identification of possible specific DMRs of inactive UC (RM)

DMRseq was performed on three individual comparisons: inactive UC (RM) vs active UC (UC), inactive UC (RM) vs controls (NN), and active UC (UC) vs controls (NN) resulting in three sets of DMRs with q < 0.05. The resulting DMRs were merged by overlapping genomic locations, and non-overlapping DMRs were discarded. To identify inactive UC specific DMRs additional t-tests were performed between the average DMR methylation levels of inactive UC samples against both control and active UC samples. DMRs whose p-value was less than 0.05 in both t-test comparisons were considered as inactive UC specific. The additional t-test ensured that the inactive UC group was in fact different from both the active UC and control group.

### Identifying DEGs correlated to specific DMRs of inactive UC (RM)

For each DMR located within the 2000 bp upstream region of the transcription start site (TSS), sample DMR and DEG were correlated using average DMR methylation and DEG expression levels by Kendall correlation^29^. UCSC known gene (GRCh38) with R package TxDb.Hsapiens.UCSC.hg38.knownGene were used for TSS sites and functional region reference^30^. Correlated genes with p < 0.1 were kept based on the correlation coefficient τ < 0.

### Annotations and pathway enrichment

Genes associated with transitional methylation patterns, where inactive UC (RM) methylation levels are between active UC (UC) and controls (NN) went through enrichment analysis for pathways enrichment using the Panther/Reactome overrepresentation analysis (Reactome version 77 released 2021–10-01) using the Fisher’s exact test^31^. For GO annotations of all 38 genes, clusterProfiler was used^32^.

### Cell deconvolutions

Cell deconvolutions were performed on DMRs that overlapped Illumina Epic array coordinates using the EpiDISH R package^33^. Average sample relative methylation values for these DMRs were used as input to EpiDISH in Robust Partial Correlation (RPC) mode. Differences between groups were calculated using ANOVA and Tukey’s range test^34^. Cell deconvolutions for the expression data were performed with CIBERSORTx (https://cibersortx.stanford.edu/). The LM22 (22 immune cell types) was selected as a signature matrix. The normalized expression matrix was chosen as the input matrix file. The remaining parameters were left at default values^35^.

## Results

### Characterisation of DNA methylation in inactive UC

By combining genome-wide methylation data and whole transcriptome data, insight into the molecular mechanisms of inactive UC was established. Bisulfite sequencing provided DNA methylation levels in patient biopsy samples from inactive UC (n = 20), treatment-naïve active UC (n = 12) and non-IBD controls (n = 11). (Table 1). The different methylation patterns between inactive UC, active UC and controls were identified by DMRseq^28^. DMRs were detected in the following comparisons: 313 DMRs were detected in inactive UC vs. controls, 5,316 DMRs were detected in inactive UC vs. active UC, and 8,262 DMRs were detected in active UC vs. controls. By considering all three comparisons, the methylation levels of a total of 3098 DMRs were negatively correlated with neighbouring transcript expression. Analysis of the combined DMRs revealed 52 DMRs (38 genes) with specific and transitional patterns as depicted in Supplementary Data 1, Fig. 2. Principal component analysis (PCA) with the correlated DMRs could discriminate samples of inactive UC, active UC, and controls at the transcriptomic level (Fig. 3), with 51.0% and 13.3% variance. An example for detailed comparisons between expression and methylation levels are visualised for annexin A11 (ANXA11) (Fig. 4). Here, the presence of DMRs provides valuable information regarding the potential regulation positions and overlapping on the promoter region (shown as “Prom” in Supplementary data 2). ANXA11 is specifically hypo-methylated in DMRs on chr10.470 compared to controls and active UC, thus the expression of ANXA11 in inactive UC is uniquely over-expressed compared to controls and UC (upper left panel, Fig. 4). It is hereby noted that the DMRs labels are specific to this study and do not represent universal IDs.

**Figure 2:**
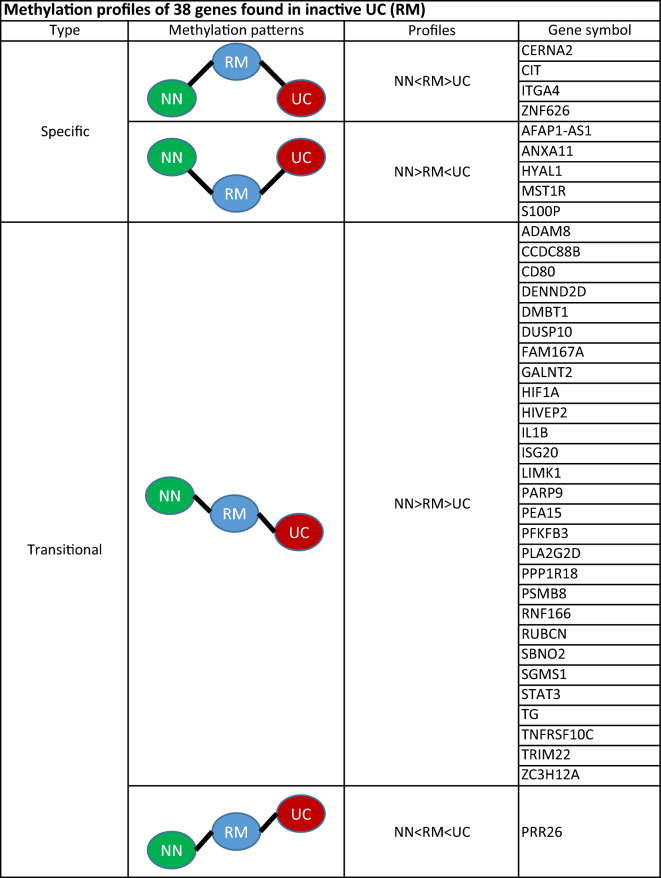
Methylation profiles of 38 DEGs found for inactive UC (RM). DEGs are assigned to specific or transitional profiles. All genes have at least one DMR which correlated to the differential expression (p.adj < 0.05) with a negative correlation p < 0.1. Patient groups representing inactive UC (RM), active UC (UC) and controls (NN) are indicated. Methylation patterns, methylation profiles, and gene symbols are indicated. A complete overview is listed in Supplementary Data 1.

**Figure 3:**
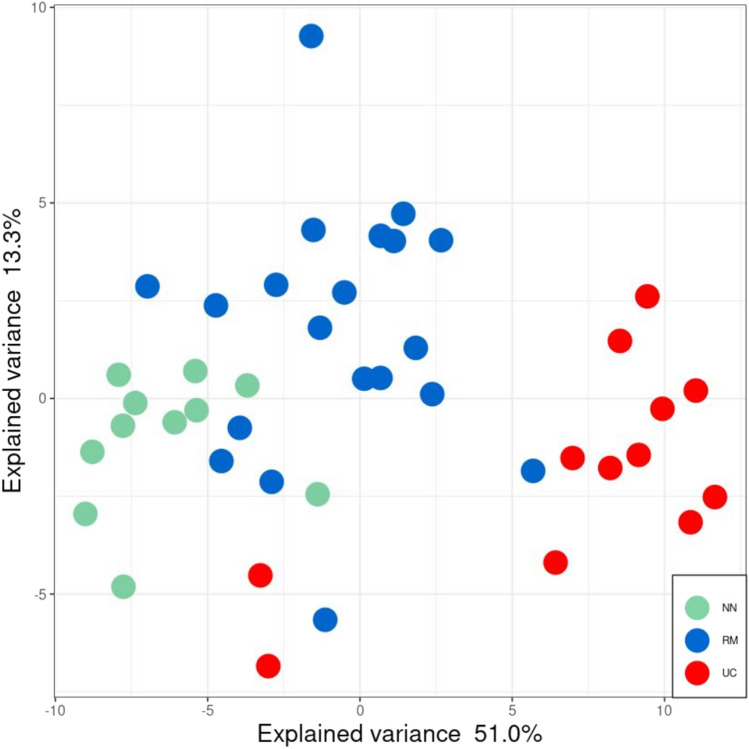
Principal component analysis (PCA). Expression levels of 38 methylation-regulated transcripts with specific and transitional patterns. Patient and control samples are indicated as followed: inactive UC (RM; blue), treatment-naïve active UC (UC; red), and controls (NN; green). Differential expression showed a 51.0% explained variance in PC1 and a 13.3% explained variance in PC2.

**Figure 4:**
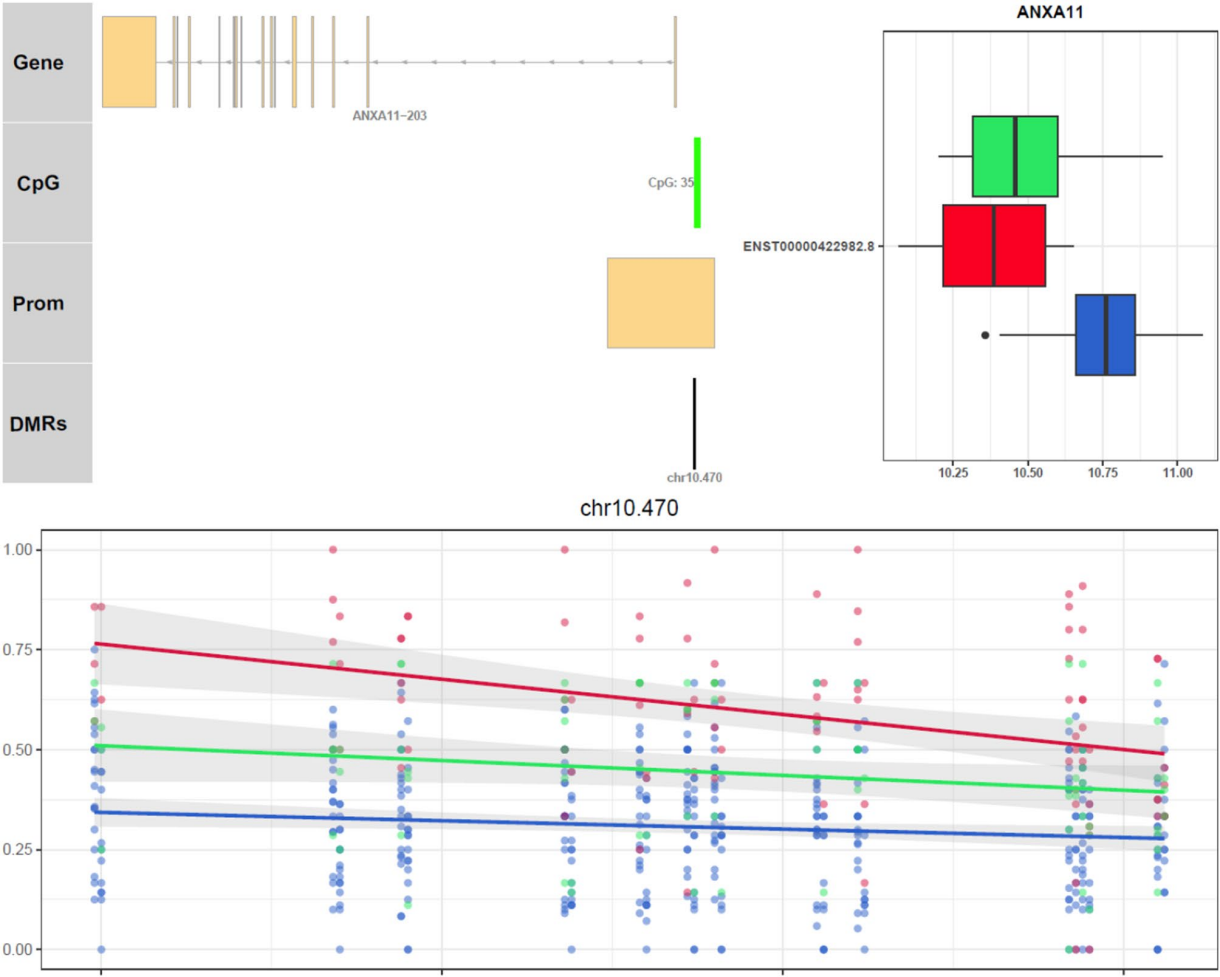
Correlation between DMRs status and transcriptional levels of ANXA11. The transcript and regulatory position of the transcript is aligned with identified DMRs on the top left, showing the regional transcript information. The data type is listed on the left: Prom stands for the promoter region in the transcript. DMRs are the differential methylated region from DMRseq, named with a chromosome and a tag. The differential expression level is shown in the top right as a box plot. The X-axis shows the log2 normalised expression levels. The differential methylation level of each region is shown at the bottom. The X-axis is the position of methylated sites in the region, and Y-axis is the methylation percentage (from 0 to 1). Each dot is one percentage of methylation position in each sample. The linear regression is shown as a line with inactive UC (blue), UC (red) and controls (green). The grey area of the line stands for a 95% confidence level. Raw difference of methylation level, differential expression level, and correlation value can be found in Supplementary Data 1. Figures of all 38 genes can be found in Supplementary Data 2, different transcripts of one gene are indicated if multiple transcripts are involved.

### Methylation-regulated gene profiling in inactive UC.

Among the 38 genes, two methylation patterns were found, one which is specific for inactive UC compared to controls and active UC, and one where methylation patterns of inactive UC are in transitional state between controls and active UC (Fig. 2). Two specific profiles were identified where inactive UC can be further subdivided into hyper- and hypomethylated compared to UC and controls. Two different transitional profiles were identified where inactive UC shows an intermediate state of methylation in relation to controls and active UC (Supplementary Data 1).

Genes with specific methylation patterns that are hyper-methylated in inactive UC include competing endogenous lncRNA 1 for mir-4707-5p and mir-4767 (CERNA2), citron rho-interacting serine/threonine kinase (CIT), integrin subunit alpha 4 (ITGA4), and zinc finger protein 626 (ZNF626). Genes uniquely hypo-methylated in inactive UC compared to active UC includes actin filament associated protein 1 antisense RNA 1 (AFAP1-AS1), annexin A11 (ANXA11), hyaluronidase 1 (HYAL1), macrophage stimulating 1 receptor (MST1R), and S100 calcium binding protein P (S100P).

The transitional genes patterns represent genes that have an intermediate methylation status between UC and controls (Fig. 2). 28 genes were found to be hyper-methylated in controls compared to active UC, including ADAM metallopeptidase domain 8 (ADAM8), coiled-coil domain containing 88B (CCDC88B), CD80, DENN domain containing 2D (DENND2D), dual specificity phosphatase 10 (DUSP10), signal transducer and activator of transcription 3 (STAT3), and interleukin 1B (IL1B), interferon stimulated exonuclease gene 20 (ISG20) and LIM domain kinase 1 (LIMK1). DIP2C antisense RNA 1 (PRR26) is the only gene found to be hyper-methylated in active UC compared to controls. A comprehensive list of figures of all methylation-regulated genes can be found in Supplementary Data 1.

### Methylation-regulated genes are related to inflammation

**Figure 5:**
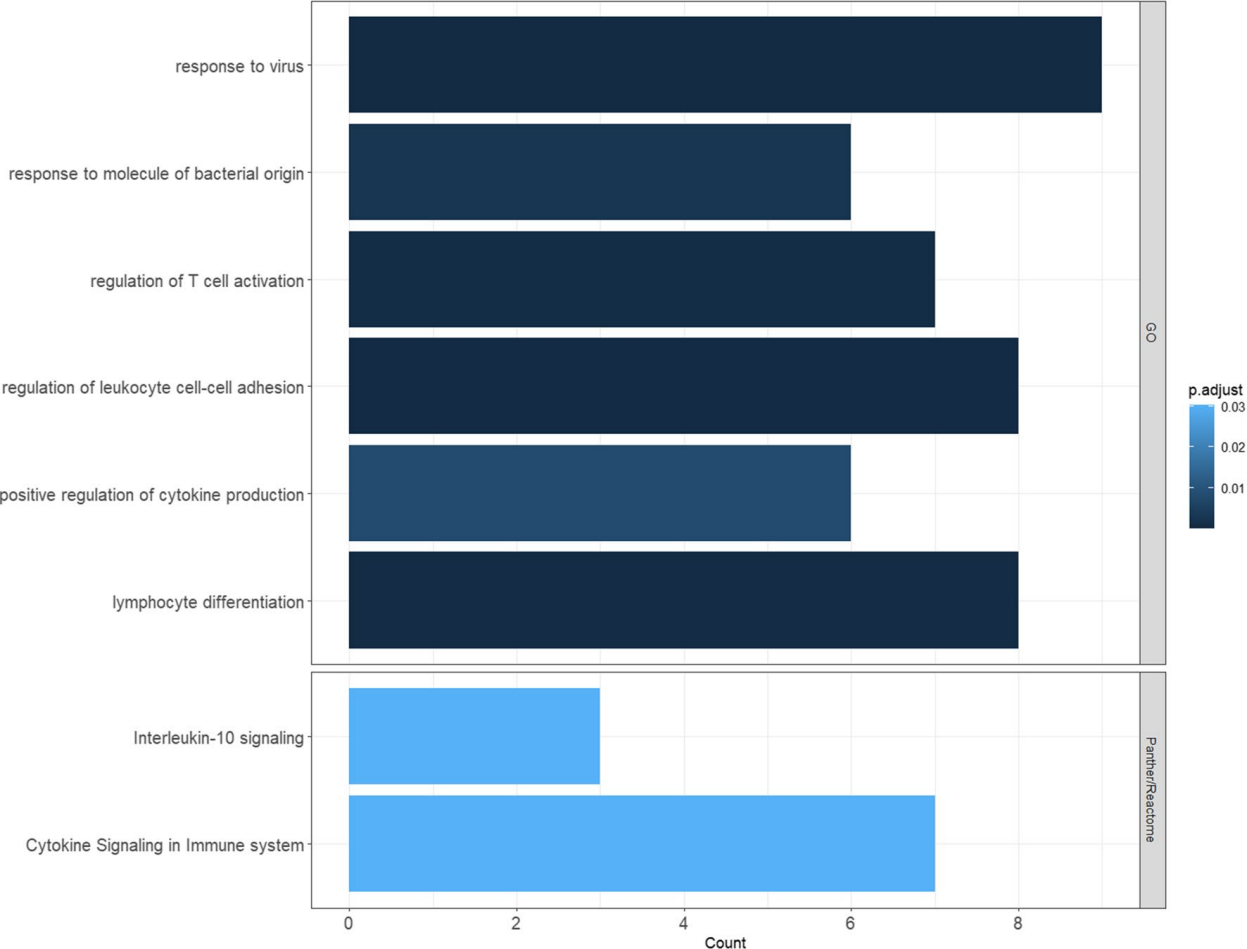
Gene ontology enrichment of the methylation correlated genes. The bar length shows the number of genes enriched in each term (x-axis). The colour stands for the padj value from dark blue to light blue (0 to 0.05, respectively). The detailed genes included in each term are listed in Supplementary Data 3.

The 28 methylation regulated genes in the intermediate state were functionally annotated with Gene Ontology (GO) terms with the Panther/Reactome overrepresentation test (Reactome v.77, released 2021-10-01) (Fig. 5). Genes like TRIM22, PSMB8, CD80, IL1B, ISG20, HIF1A, STAT3 were all hyper-methylated and downregulated to a lesser extent in inactive UC compared to UC. These genes were annotated to the IL-10 pathway and cytokine signalling in immune system (Fig. 5).

Functional enrichment revealed 101 immunological and inflammation related signalling pathways (p.adj < 0.05), which include interleukin-6 production, CD4-positive, alpha–beta T cell activation and lymphocyte differentiation represented by genes like ADAM8, IL1B, ISG20, CD80, STAT3 and ZC3H12A (Fig. 5; Supplementary Data 3). Genes in specific patterns including CIT, MST1R, HYAL1, ITGA4 were annotated as hyaluronan metabolic process, epithelium migration and phagocytosis. The total of 103 functional GO terms are listed in Supplementary Data 3.

### Cell deconvolutions based on DNA methylations discriminate cell fractions

**Figure 6:**
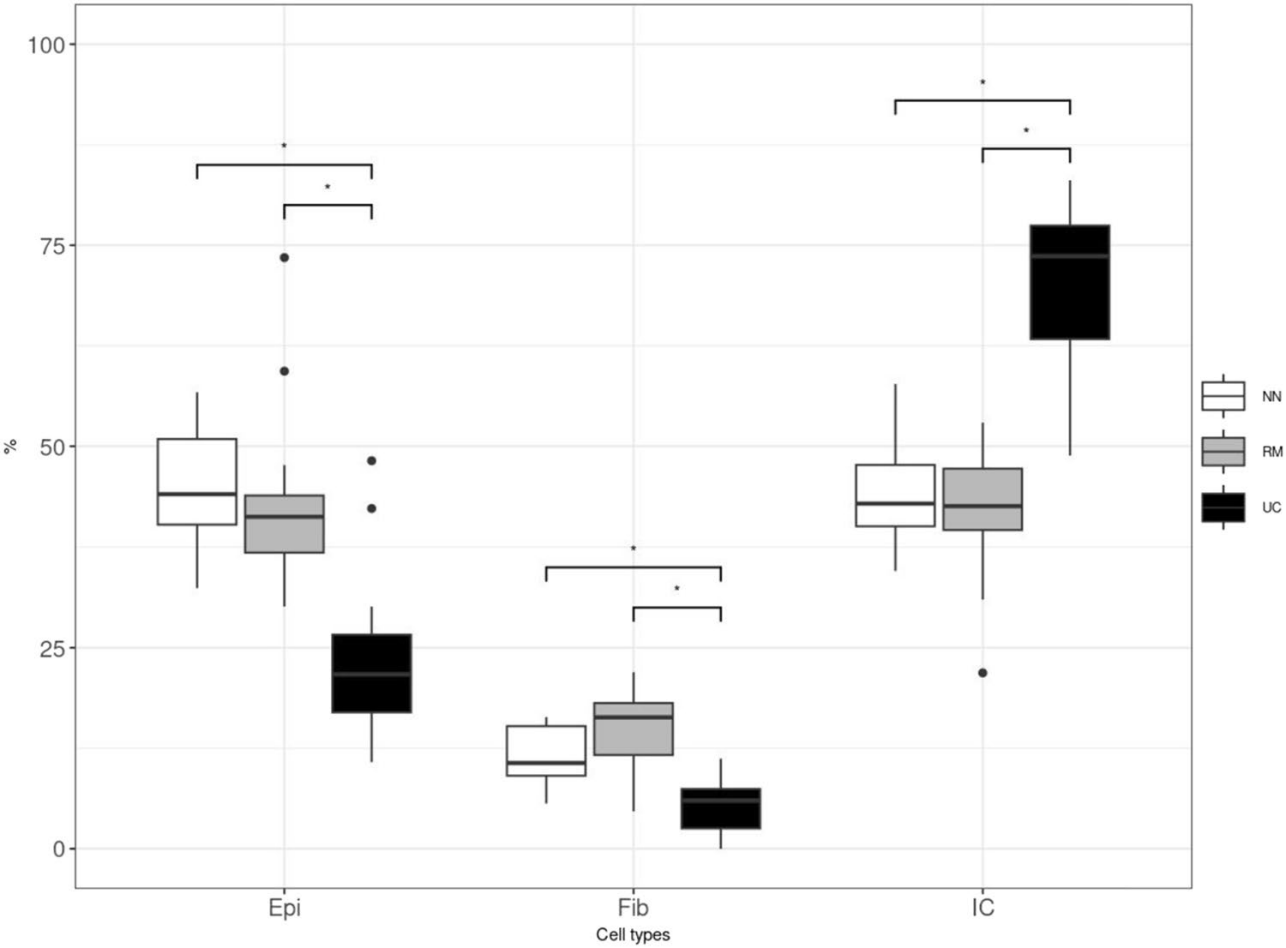
Cell deconvolutions. The cell types are shown in X-axis as Epi (epithelium), Fib (fibroblasts) and IC (immune cells). The percentages of the cell types are shown on the Y-axis.

Cell deconvolution was performed by mapping DMR genomic coordinates to Illumina EPIC array identifiers^33^. The deconvolution results revealed differences in cell fractions for inactive UC (RM), active UC (UC) and controls (NN). Epithelial cell fractions were higher in normal and inactive UC than active UC (padj < 0.01). The proportion of immune cells is significantly higher in active UC compared to inactive UC and controls (padj < 0.01). Similarly, the fibroblast cell fractions in inactive UC and controls are slightly higher than in active UC (Fig. 6, Supplementary Data 4). Using CIBERSORTx on the normalized gene expression matrix showed an increase in most immuno-derived cells, especially of neutrophils in active UC as compared to inactive UC and controls (Supplementary Data 5)^35^.

## Discussion

In a previous work, DNA methylation patterns have been identified for active, treatment-naïve UC^12,13,36^. In this study, the methylation status in inactive UC (RM) has been explored by determining gene expression regulated by global DNA methylation overlapping the promoter region of genes. By correlating DNA methylation data to expression levels of genes several regulatory DNA methylation features of relevance for inactive UC could be identified and are discussed below.

Long-term treatment of UC patients is often necessary to achieve induction and maintenance of clinical remission^16–18^. UC medications such as immunosuppressive drugs have been shown to have side effects on immune response and can change the DNA methylations status^37,38^. Induced epigenetic changes due to long-term treatment can be expected for 5-ASA which is commonly used as a first-line treatment for UC and might therefore have the potential to change the methylation status. This notion is supported by in vitro studies that have shown that 5-ASA treatment increases the expression of DNA methyltransferase 1 (DMNT1) which is responsible for most of the methylation events occurring on the human genome^39^ Therefore, it can be anticipated that the observed methylation changes in inactive UC patients might be a result of 5-ASA treatment.

Four genes have been found to be specifically hyper-methylated in inactive UC compared to active UC and controls (NN) (Fig. 2), CERNA2, CIT, IGTA4 and ZNF 626 (Supplementary Data 1). The observed specific hyper-methylation of CERNA2 in inactive UC compared to UC indicates anti-inflammatory characteristics. CERNA2 has been recently reported to play a role in inflammation^40^ and has been identified as an independent predictor for clinical prognosis of gastric cancer^41^. It is notable that the expression of CERNA2 has been shown to correlate with poor clinical parameters and an unfavourable prognosis of different cancer patient groups while silencing of CERNA2 expression inhibits cancer cell growth and promotes cell apoptosis^42,43^. In this context, CIT was specifically hyper-methylated in inactive UC. Silencing of CIT has been shown to reduce tumour growth in multiple myeloma and breast cancer cells by promoting apoptosis^44,45^. Therefore, specific hypermethylation of CIT and CERNA2 might play a role in the fine-tuning of the regulation of apoptotic events during inactive UC^21^. ITGA4 is another specifically hyper-methylated gene in inactive UC compared to UC and is a well-known therapeutic target for the treatment of IBD (Fig. 2 & Supplementary Data 2). The observed downregulation of ITGA4 expression implies reduced leukocyte infiltration into the GI tract through the interaction with MAdCAM‐1 which is expressed on high endothelial venules (HEV) within vessels of mucosal tissue^46–50^.

Quiescent inflammation present in inactive UC has previously been reported^20,51,52^ and many of the observed methylation-regulated expressed genes identified in inactive UC are involved in inflammation. Surprisingly, increased levels of a well-known marker for inflammation S100P were observed to a greater extent in inactive UC compared to active UC and controls. The observed specific hypo-methylation and upregulation of S100P in inactive UC might, in addition to S100P’s inflammatory responses, contribute to the regulation of tissue development and regeneration or repair as previously reported^53^ (Fig. 2 & Supplementary Data 2).

Chronic inflammation like UC results in dramatic deposition of hyaluronic acid (HA) within affected tissues which both precedes and promotes immune cell infiltration, tissue destruction, and coagulation^54^. The observed increased expression of HYAL1 in inactive UC might lead to decreased levels of HA in active UC, thereby modulating the promotion and resolution of the disease by controlling recruitment of immune cells, by release of inflammatory cytokines, and by balancing haemostasis^55^ (Supplementary Table 2,Fig. 2). Hypo-methylation and increased expression of MSTR-1 in inactive UC compared to UC might directly be involved in the wound healing process by promoting epithelial cell migration and proliferation, as this innate immune response regulates the migration of macrophages increasing the phagocytic activity^56^. Increased fractions of epithelial cells and decreased fractions of immune cells in inactive UC are in concordance with the results obtained by cell deconvolutions (Fig. 6).

Two other specifically hypo-methylated genes in inactive UC of relevance for IBD include ANXA11 and lncRNA AFAP1-AS1. ANXA11 has been shown to be a sarcoidosis susceptibility gene^57^. An association between sarcoidosis and ulcerative colitis has been reported^58^. The expression of lncRNA AFAP1-AS1 has been shown to promote the progression of CRC^59^ and has been acknowledged as a biomarker for diagnosis and prognosis estimation of CRC patients^60^. However, the potential role of the here observed hypo-methylation of AFAP1-AS in inactive UC is still unclear.

Annotations of genes with intermediate methylation patterns for inactive UC revealed two pathways, IL10 signalling and cytokine signalling in immune system pathways (Fig. 5, Supplementary Data 3). These genes were all hyper-methylated and downregulated in inactive UC compared to active UC but to a lesser extent then in controls, meaning that normal levels of expression are not completely achieved by hyper-methylation. The downregulation of the proinflammatory cytokine IL1B, might reduce T cell immune response by downregulation of co-stimulatory molecules such as CD80^61–63^. In addition, IL1B production is diminished by the observed downregulation of STAT3 expression and implies reduced phosphorylation of IL1B. Hyper-methylation of STAT3 in inactive UC compared to active UC might be involved in the regulation of adaptive immune responses by reducing survival of pathogenic T cells and TNF-α^64^. The transcriptional coregulator SBNO2 (strawberry notch homolog 2) is hyper-methylated in inactive UC compared to UC and contributes to the downstream anti-inflammatory effects of IL-10 which is dependent on STAT3 activation^65^.

Compared to controls, ZC3H12A is hypo-methylated in inactive UC which might indicate a modulation of the inflammatory response by promoting the degradation of a set of translationally active cytokine-induced inflammation-related mRNAs, such as IL6 and IL12B^66^. ZC3H12A induces the deubiquitylation of the transcription factor HIF1A which is also hyper-methylated in inactive UC compared to active UC, thereby positively regulating the expression of proangiogenic HIF1A-targeted genes^67^. The decrease of HIF1A expression in inactive UC may function as a transcriptional regulator of the adaptive response to hypoxia maintaining biological homeostasis^68^. In this context, cell deconvolutions revealed epithelial cell fractions in inactive UC were comparable to epithelial fractions in control samples (Fig. 6). It is notable that somatic mutations of ZC3H12A have been found in UC patients’ epithelium which might have an unknown influence on the DNA methylation regulated expression^69^. The hypo-methylation of LIMK1 in inactive UC compared to controls could lead to reduced T-cell regulation in inactive UC through Rho/Rac pathways. A single nucleotide polymorphism (SNP) rs6460071 in LIMK1 has been reported to be most significantly associated with proximal endoscopic extension in CRC and is a predictor of outcome in UC^70^.

DNA methylation has been found to influence the regulation of interferon’s antiviral processes mediated by TRIM22, IGS20, and DENND2D. The hypermethylation of the interferon-induced antiviral protein TRIM22 compared to UC might contribute to a decrease in disease development through the NF‑κB signalling pathway^71^. The increased expression of antiviral ISG20 in UC still needs to be confirmed. However, the hypo-methylation and increased expression of ISG20 in inactive UC compared to controls may be a potential susceptibility biomarker or pharmacological target as has been shown for other inflammatory conditions^72^. It is hereby noted that a prognostic impact of expression and methylation status of DENN/MADD domain-containing protein 2D in gastric cancer has been proposed^73–75^. GALNT2 catalyses the initial reaction in O-linked oligosaccharide biosynthesis and has a broad spectrum of substrates for peptides such as MUC5AC, MUC1A, MUC1B. An increase of GALNT2 expression has been recently reported for UC patients in the active stage compared to patients in the remission^76^. This result might be in part also be explained and confirmed by the observed hyper-methylation of GALNT2 in inactive UC compared to UC (Supplementary Data 1, Fig. 2).

A limitation of this work is the small sample size and the heterogeneity of tissue samples as it was not possible to discriminate inactive UC in terms of remission duration and DNA methylation as has been recently seen for gene expression^21^. It is important to note that epigenome-wide association studies do not always precede changes in transcription as has been recently reported^77^.

## Conclusions

Several differentially expressed genes involved in IL-10/cytokine signalling pathways may be under the control of DNA methylation events which might indicate fine-tuned processes regulating the balance between quiescent inflammation and mucosal healing in inactive UC.

### Supplementary Information

Supplementary Information 1.

Supplementary Information 2.

Supplementary Information 3.

Supplementary Information 4.

Supplementary Information 5.

## Data Availability

Processed RNA-seq data are deposited in NCBI’s Gene Expression Omnibus (GEO, https://www.ncbi.nlm.nih.gov/geo/) and are accessible through GEO series accession numbers GSE128682 and GSE169360. Regarding the availability of DNA data, it is hereby noted that, according to the Norwegian Health Research Act §34, the processing of health information can only take place in accordance with the consent given. In this case, the availability of unprocessed DNA information would not be in accordance with the participants’ consent. All data generated or analysed during this study are included in this published article and supplementary data files.
