# Supplementary material for: DNA methylation fine-tunes pro-and anti-inflammatory signalling pathways in inactive ulcerative colitis tissue biopsies
Source: Sci Rep. 2024 Mar 21;14:6789. doi: 10.1038/s41598-024-57440-0 (PMC10957912; doi:10.1038/s41598-024-57440-0)
Supplement: Supplementary file 2 — Supplementary Information 2. [file 41598_2024_57440_MOESM2_ESM.pdf]

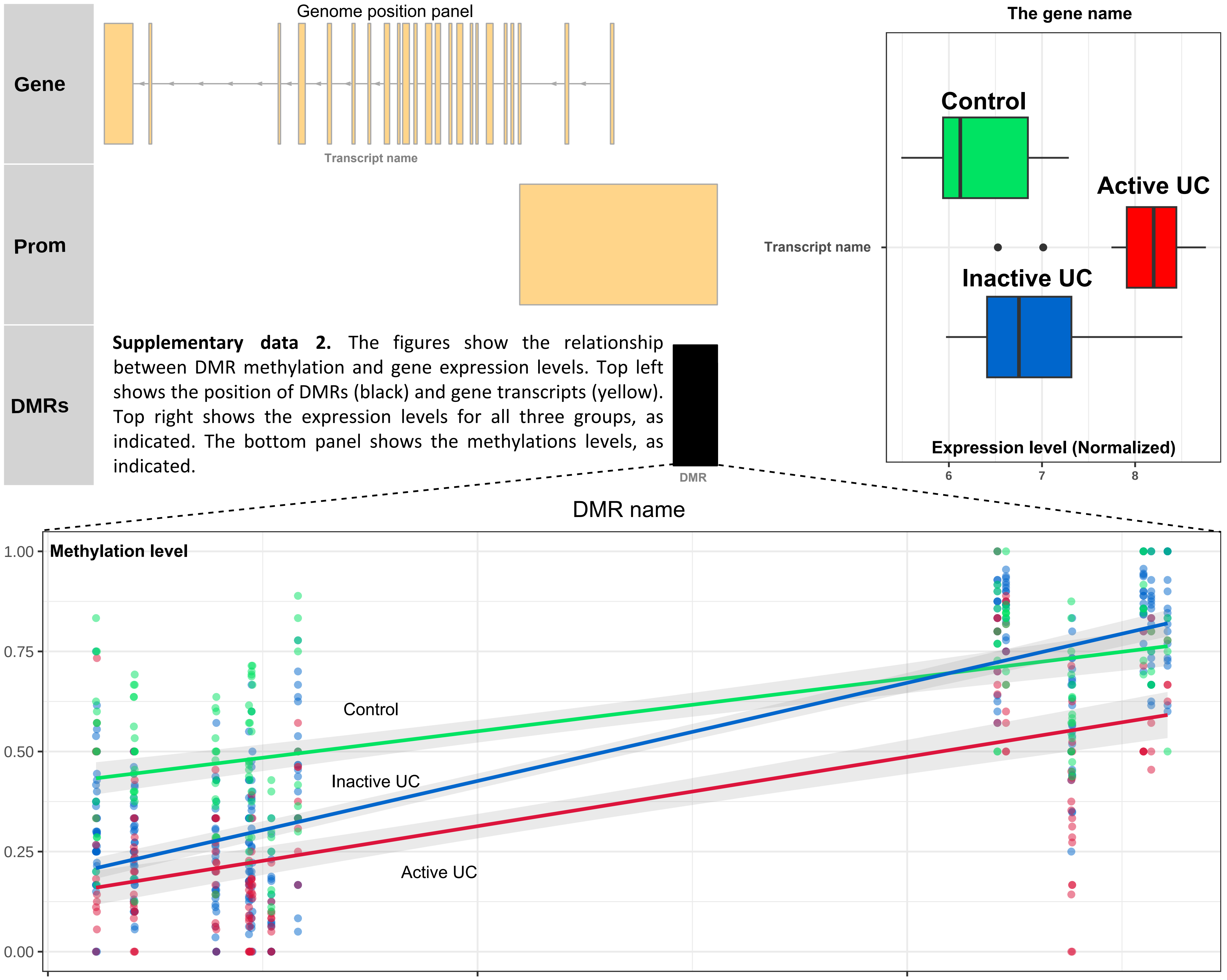

Supplementary data 2. The figures show the relationship between DMR methylation and gene expression levels. Top left shows the position of DMRs (black) and gene transcripts (yellow). Top right shows the expression levels for all three groups, as indicated. The bottom panel shows the methylations levels, as indicated.

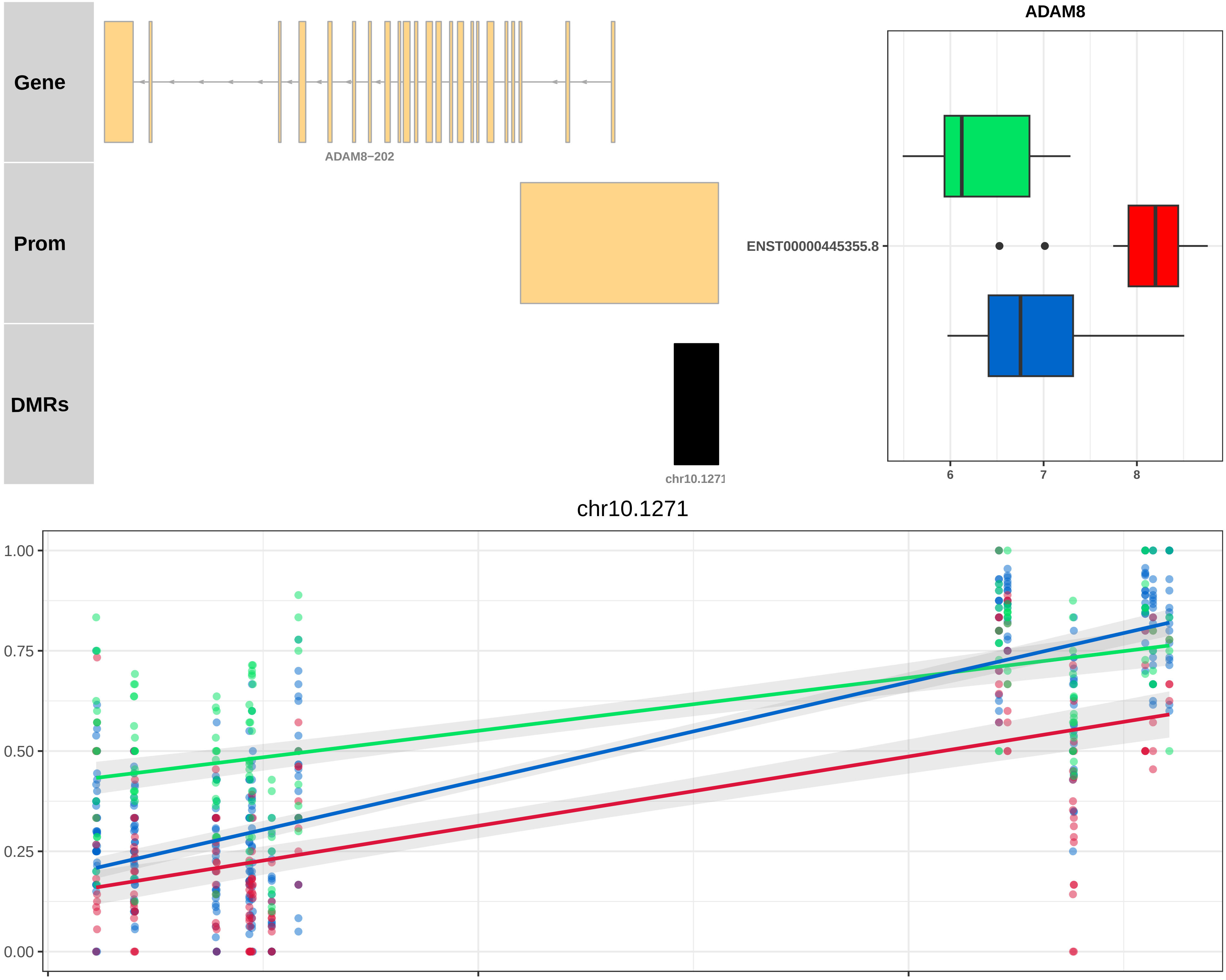

Gene
Prom
DMRs
ADAM8−202
chr10.1271
ADAM8
ENST00000445355.8
6
7
8
chr10.1271
1.00
0.75
0.50
0.25
0.00

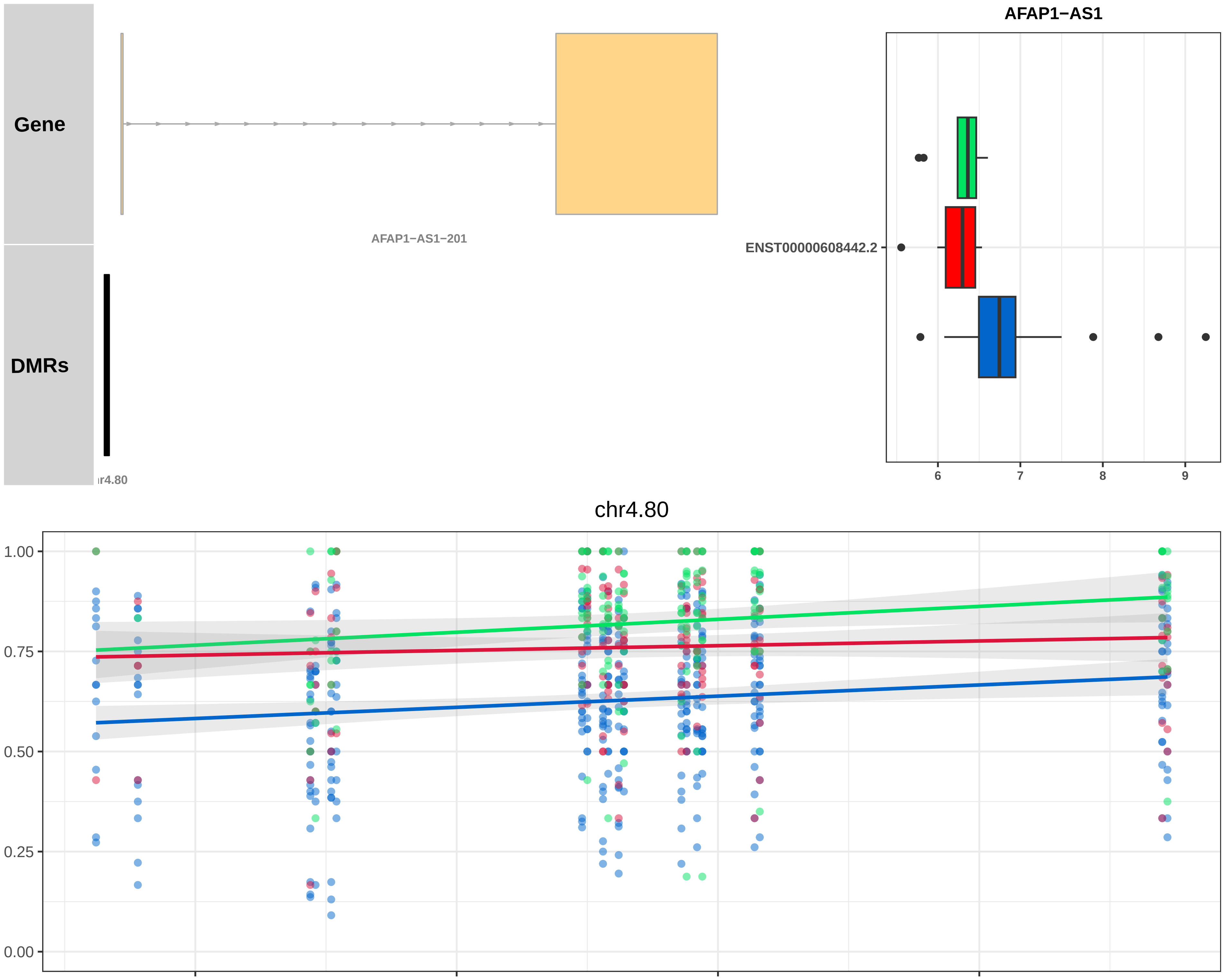

Gene
DMRs
AFAP1−AS1−201
r4.80
AFAP1−AS1
ENST00000608442.2
6
7
8
9
chr4.80
1.00
0.75
0.50
0.25
0.00

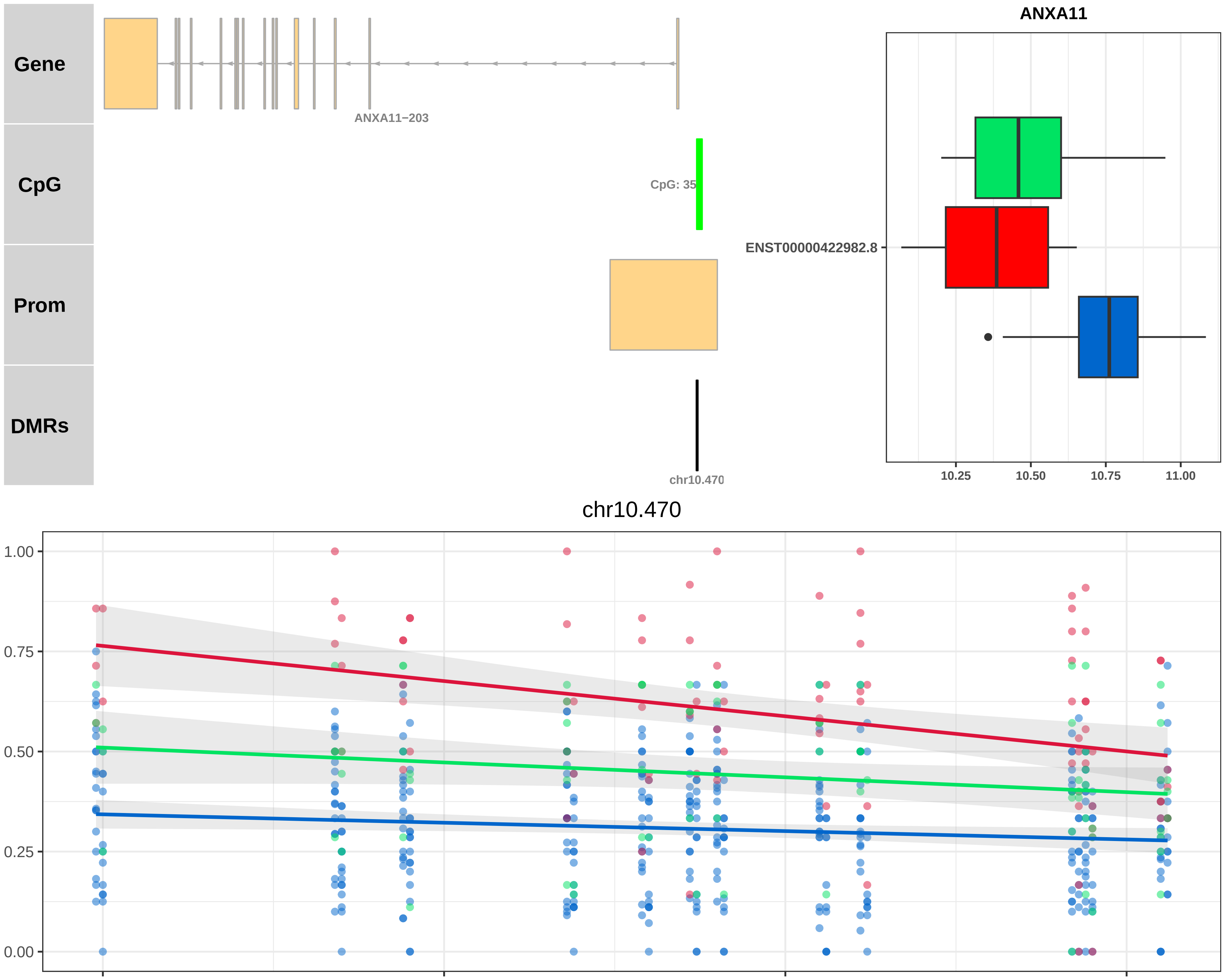
Gene
CpG
Prom
DMRs
ANXA11−203
CpG: 35
chr10.470
ANXA11
ENST00000422982.8
10.25
10.50
10.75
11.00
chr10.470
1.00
0.75
0.50
0.25
0.00

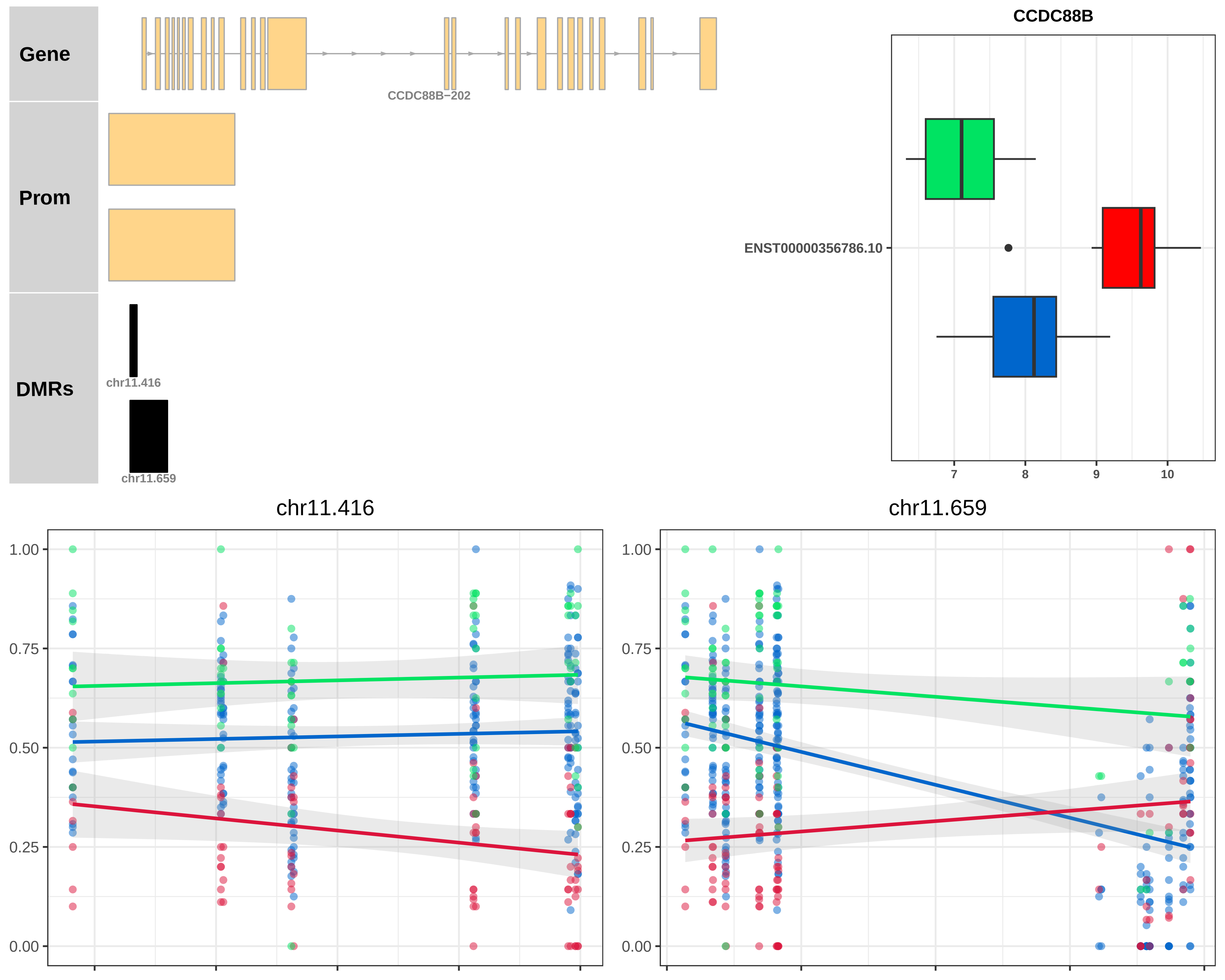
Gene
Prom
DMRs
CCDC88B−202
chr11.416
chr11.659
CCDC88B
ENST00000356786.10
7
8
9
10
chr11.416
chr11.659
1.00
0.75
0.50
0.25
0.00

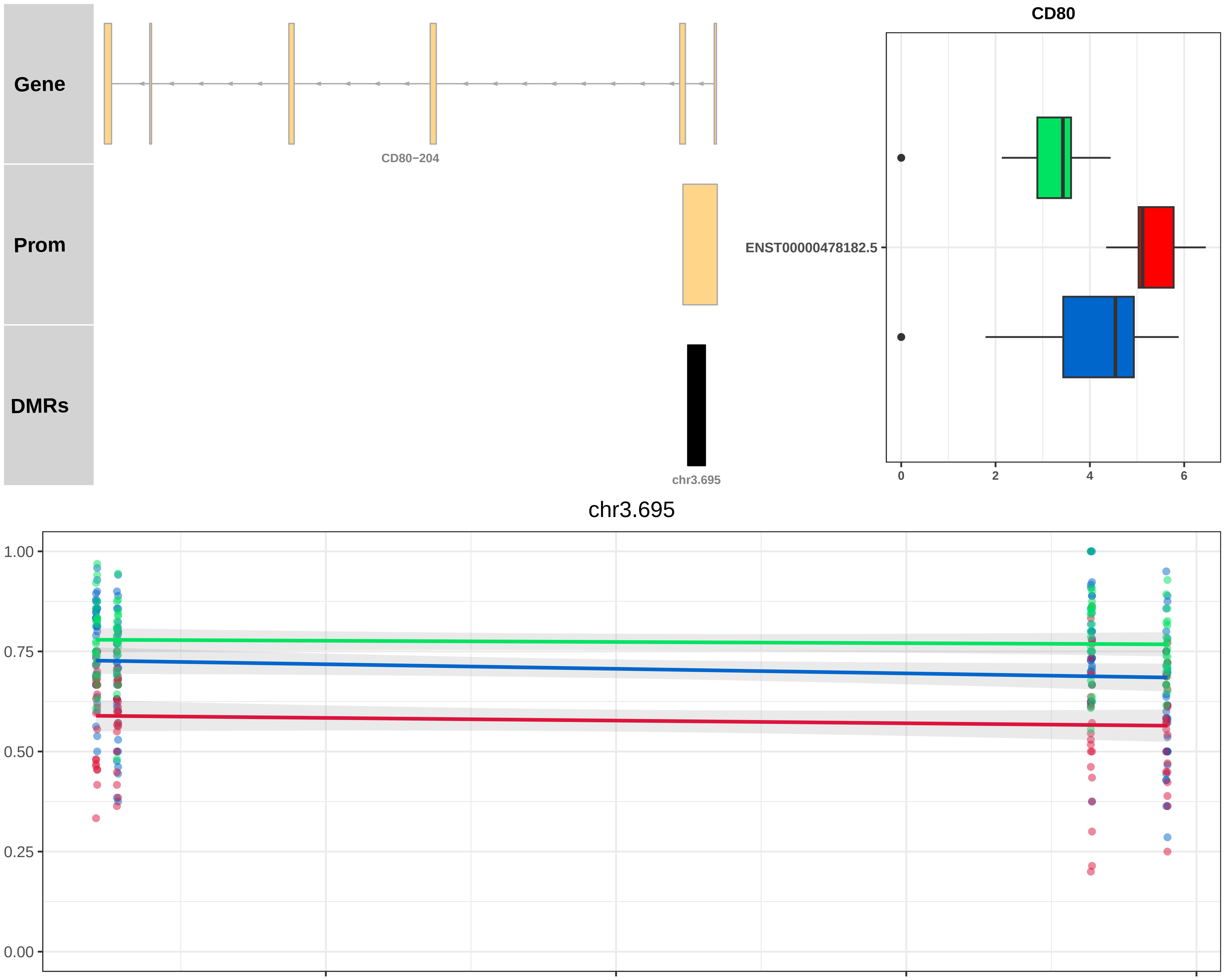
Gene
Prom
DMRs
CD80−204
chr3.695
CD80
ENST00000478182.5
0
2
4
6
chr3.695
1.00
0.75
0.50
0.25
0.00

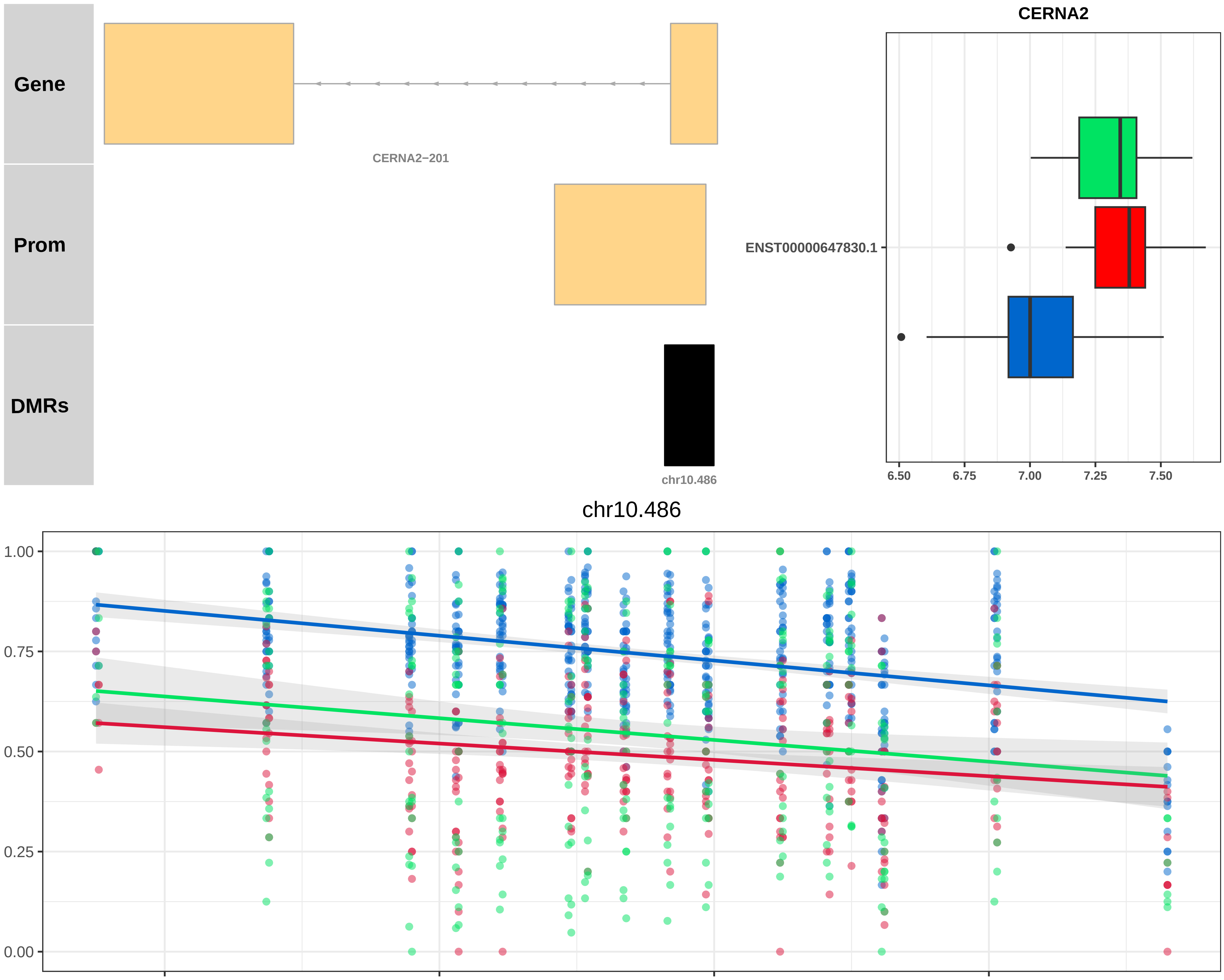

Gene
Prom
DMRs
CERNA2−201
chr10.486
CERNA2
ENST00000647830.1
6.50
6.75
7.00
7.25
7.50
chr10.486
1.00
0.75
0.50
0.25
0.00

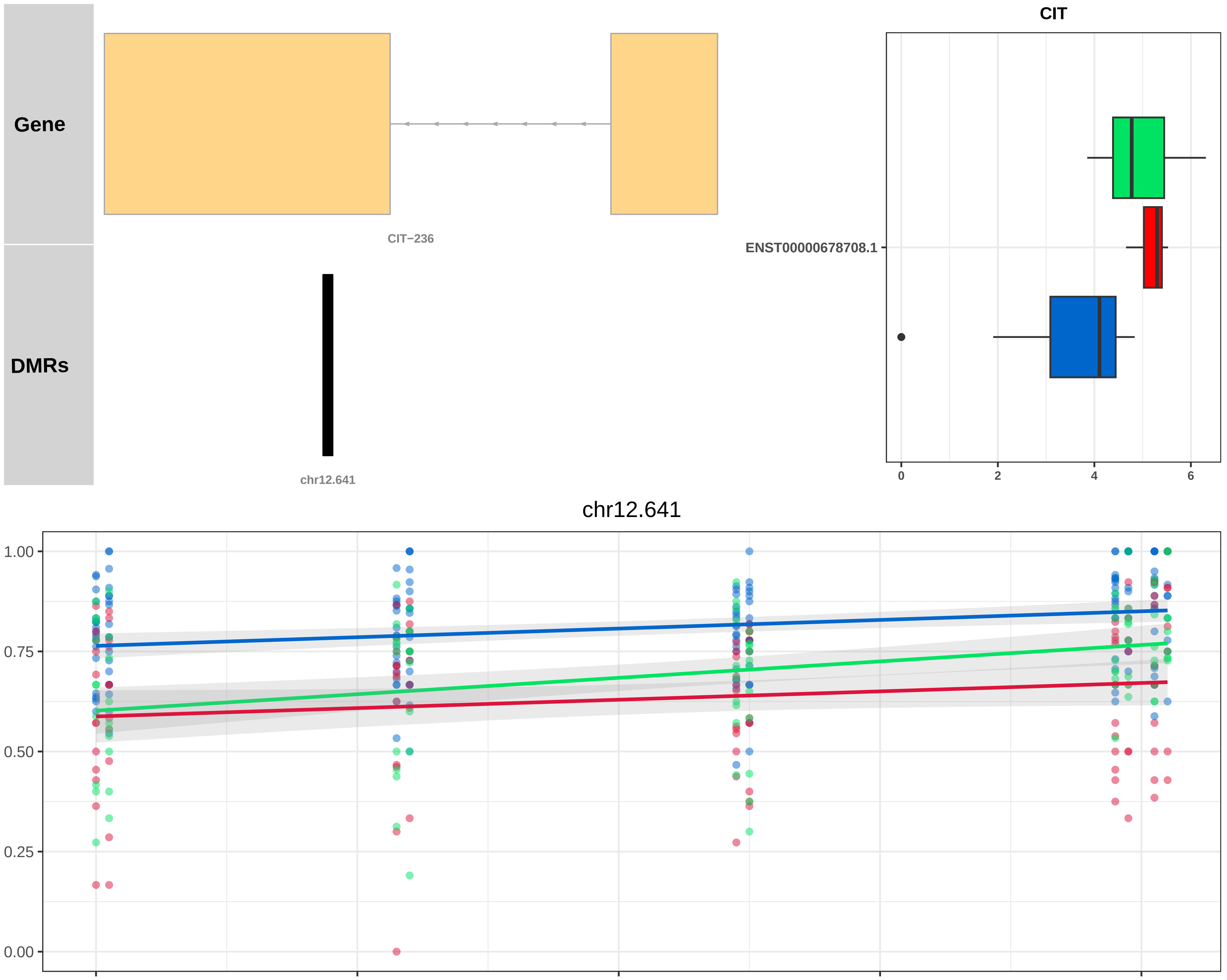

Gene
DMRs
CIT−236
chr12.641
CIT
ENST00000678708.1
0
2
4
6
chr12.641
1.00
0.75
0.50
0.25
0.00

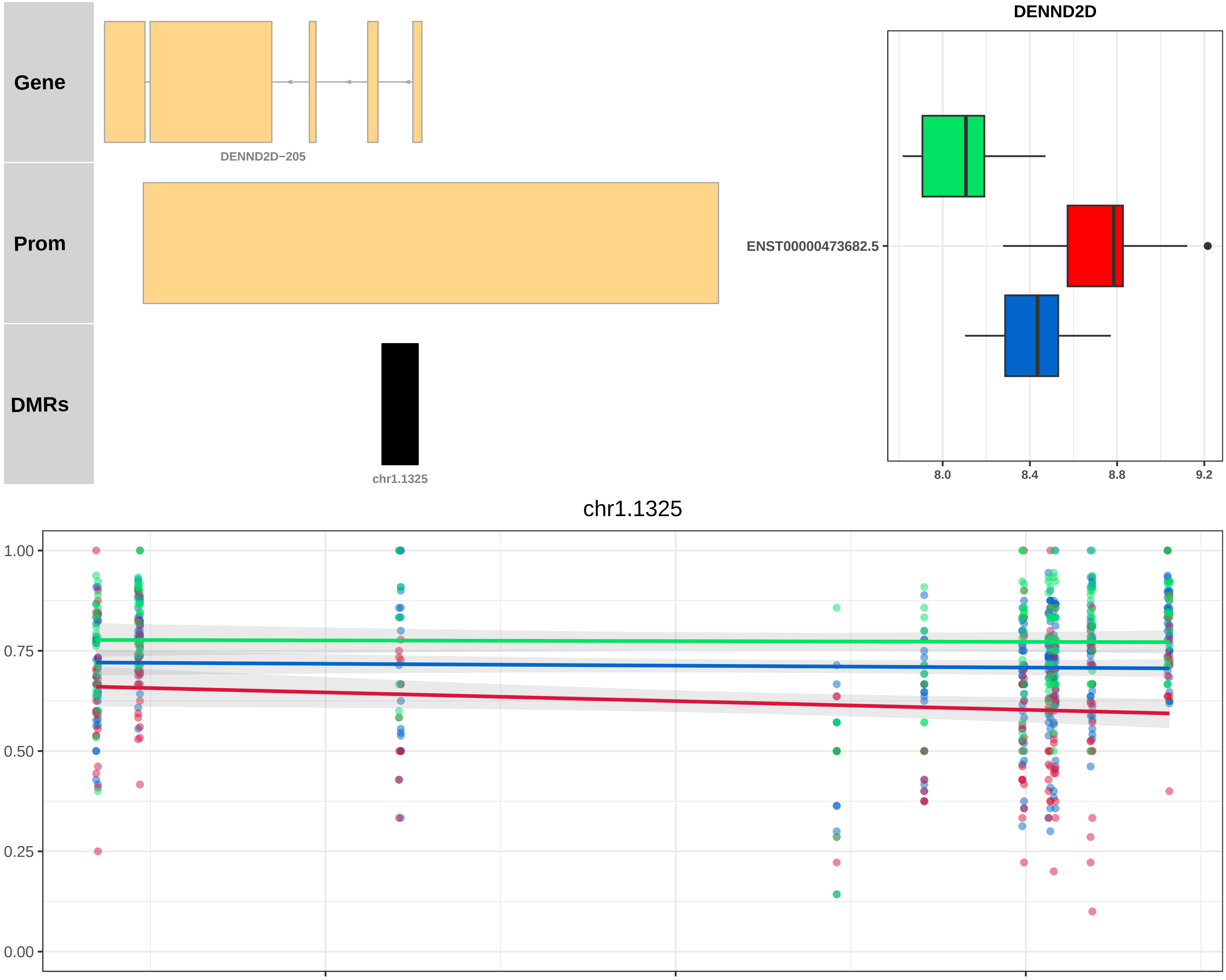
Gene
Prom
DMRs
DENND2D−205
chr1.1325
DENND2D
ENST00000473682.5
8.0
8.4
8.8
9.2
chr1.1325
1.00
0.75
0.50
0.25
0.00

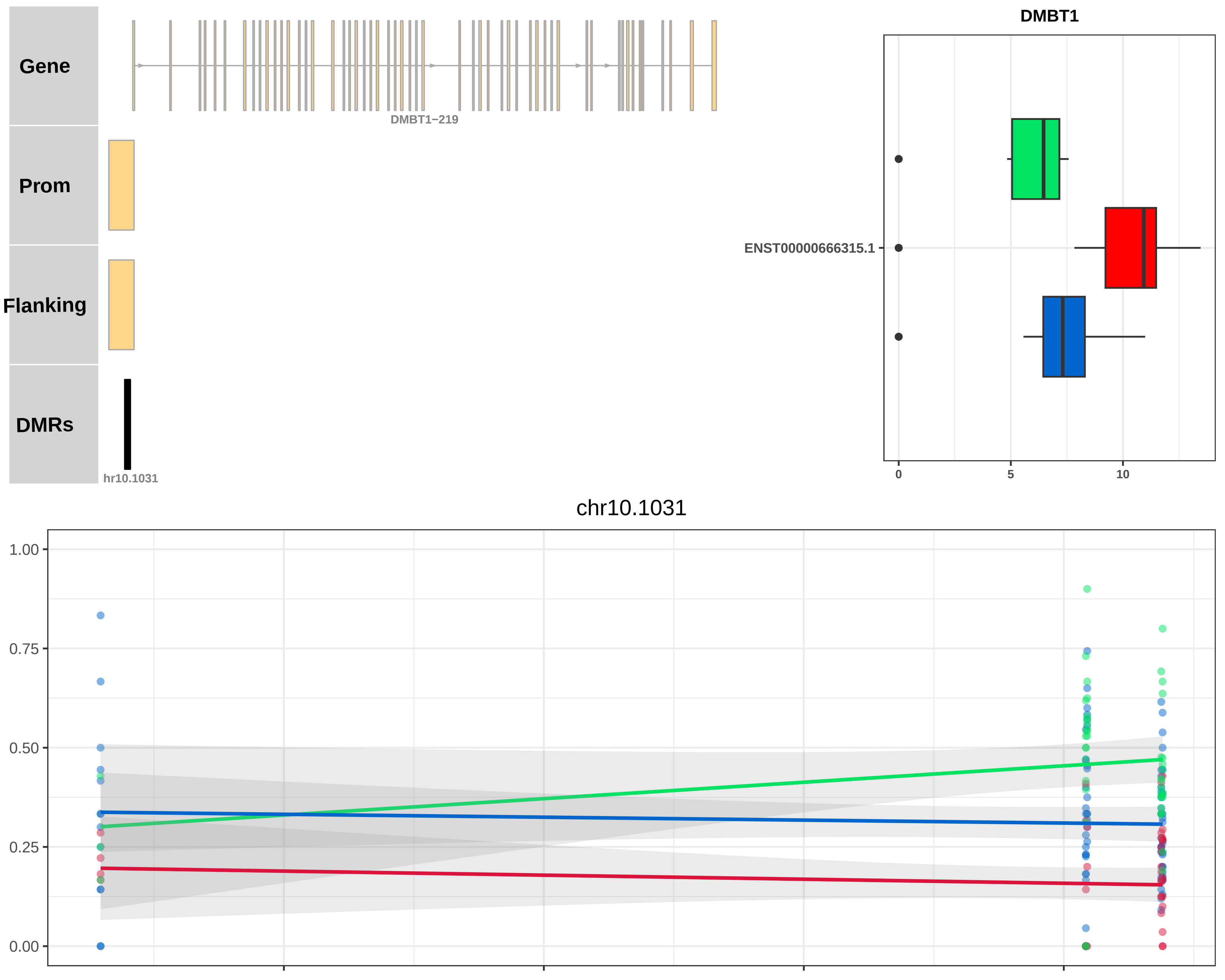

Gene
Prom
Flanking
DMRs
DMBT1−219
hr10.1031
DMBT1
ENST00000666315.1
0
5
10
chr10.1031
1.00
0.75
0.50
0.25
0.00

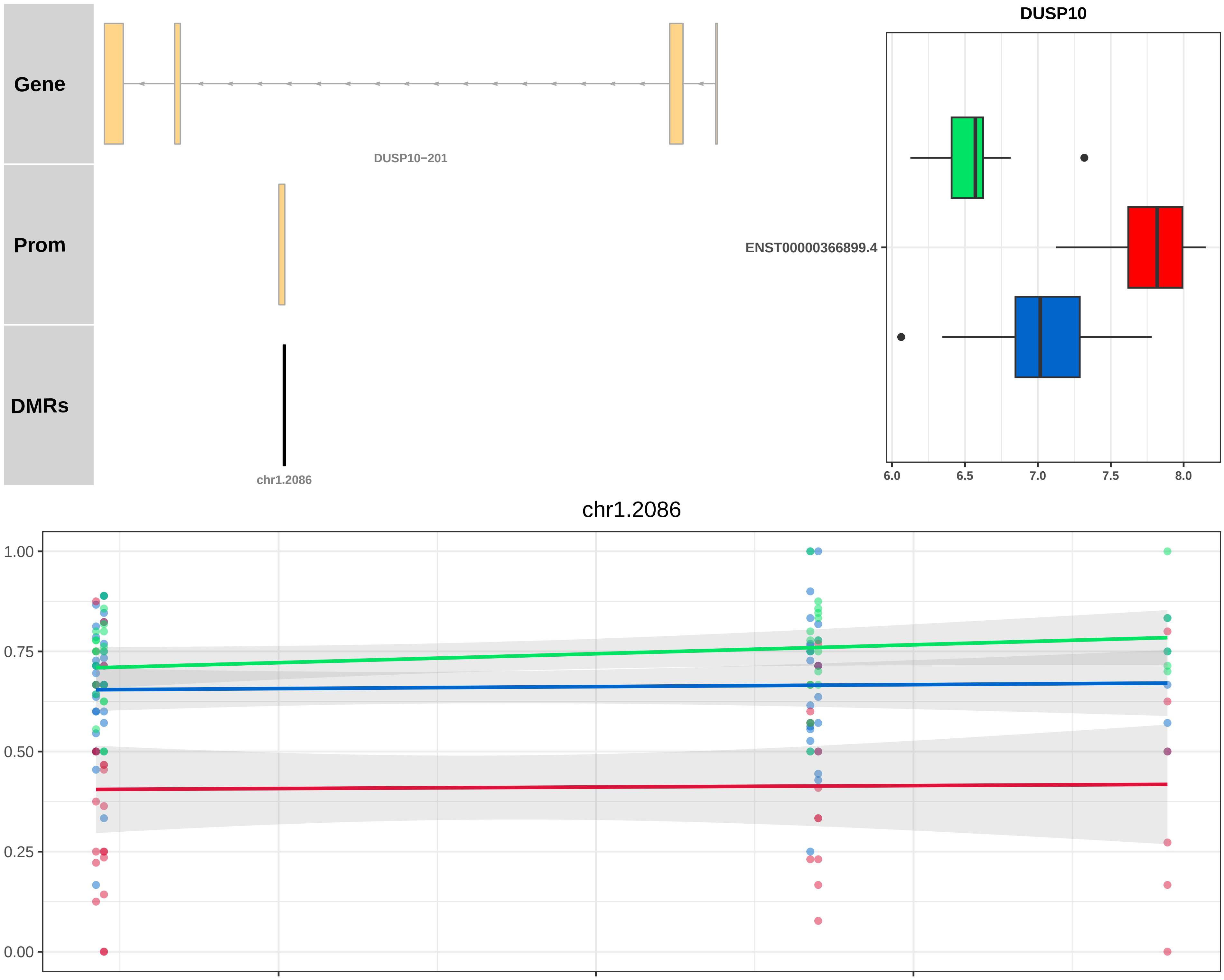

Gene
Prom
DMRs
DUSP10−201
chr1.2086
DUSP10
ENST00000366899.4
6.0
6.5
7.0
7.5
8.0
chr1.2086
1.00
0.75
0.50
0.25
0.00

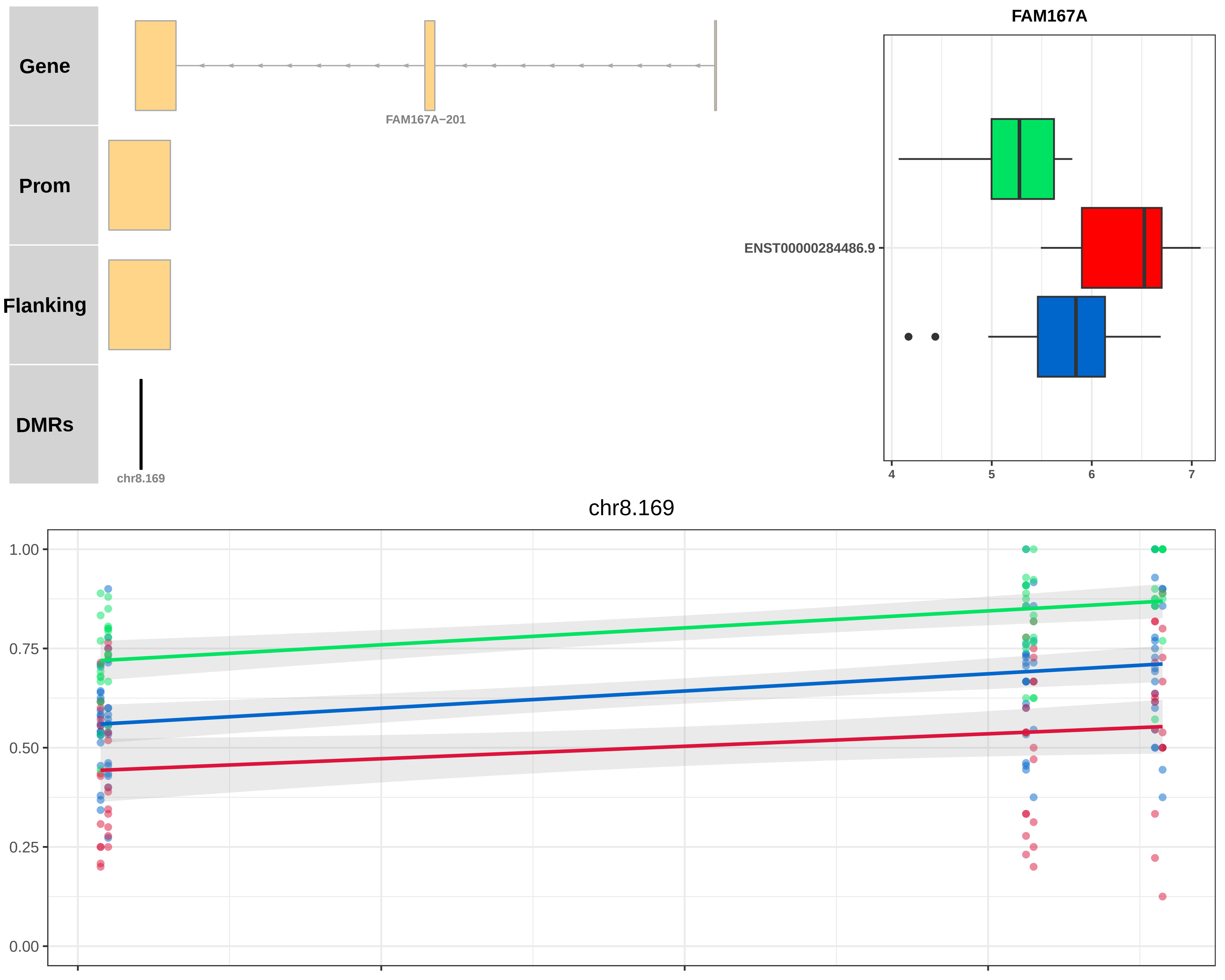

Gene
Prom
Flanking
DMRs
FAM167A−201
chr8.169
FAM167A
ENST00000284486.9
4
5
6
7
chr8.169
1.00
0.75
0.50
0.25
0.00

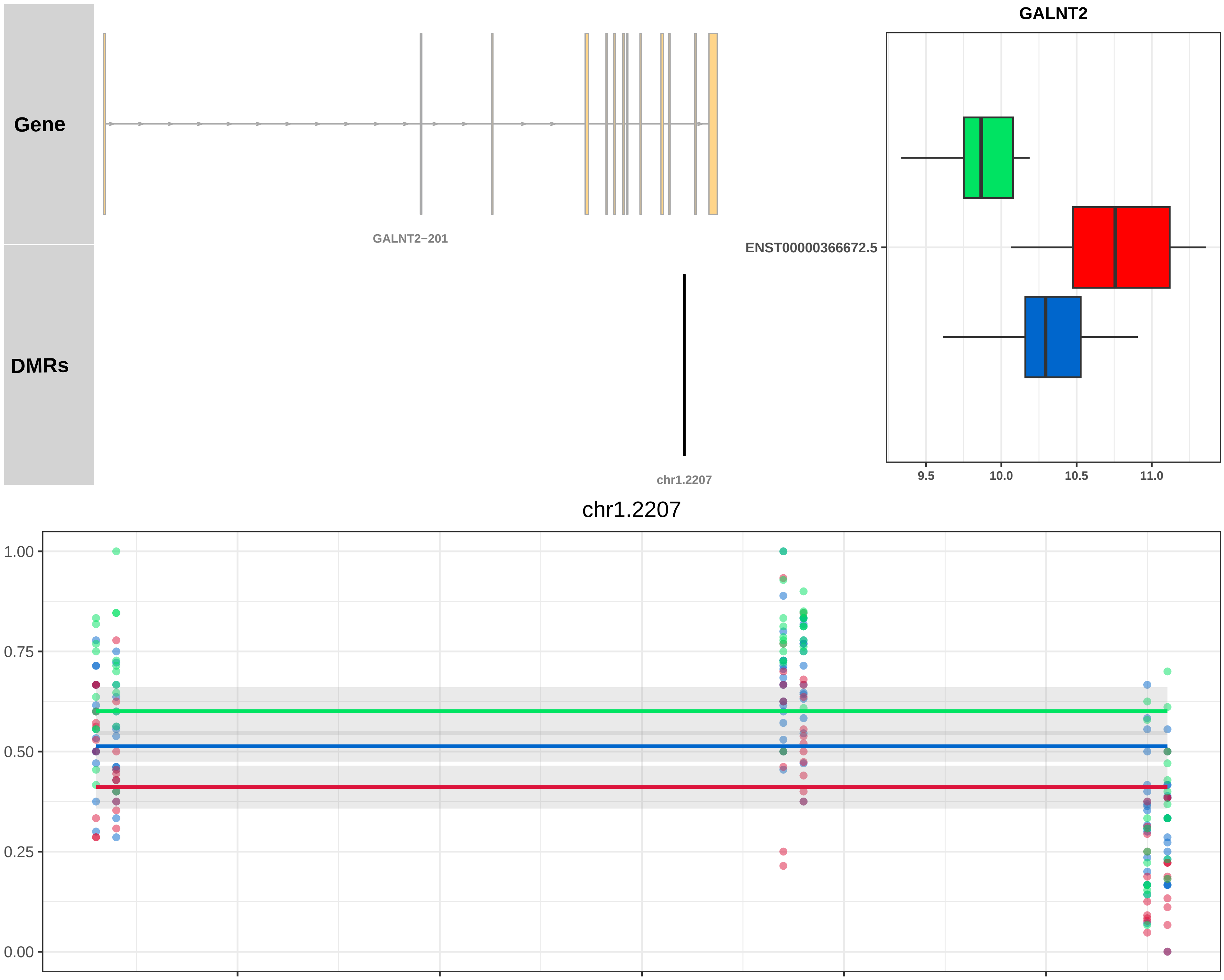

GALNT2
Gene
DMRs
GALNT2−201
chr1.2207
ENST00000366672.5
9.5
10.0
10.5
11.0
chr1.2207
1.00
0.75
0.50
0.25
0.00

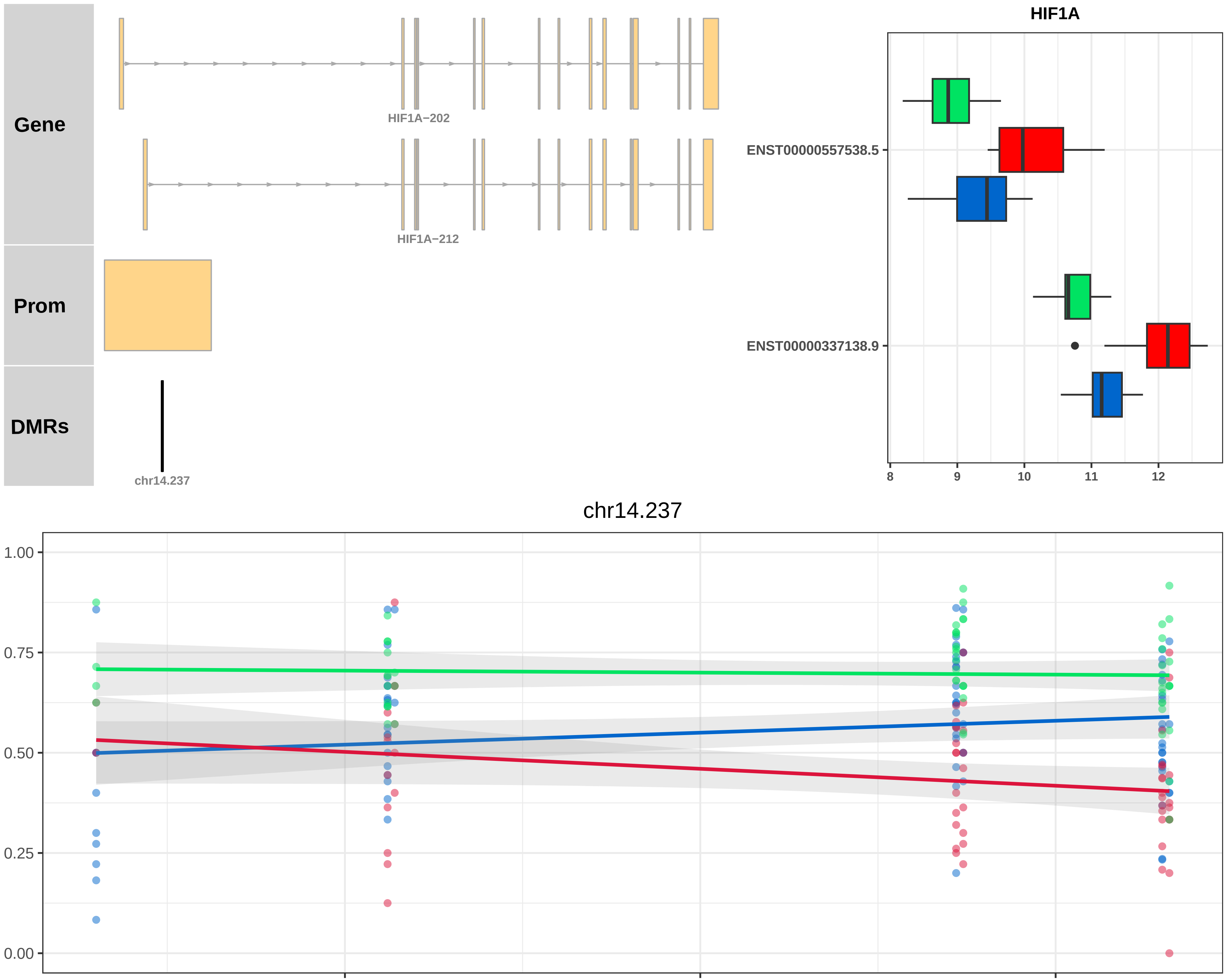

Gene
HIF1A−202
HIF1A−212
Prom
DMRs
chr14.237
HIF1A
ENST00000557538.5
ENST00000337138.9
8
9
10
11
12
chr14.237
1.00
0.75
0.50
0.25
0.00

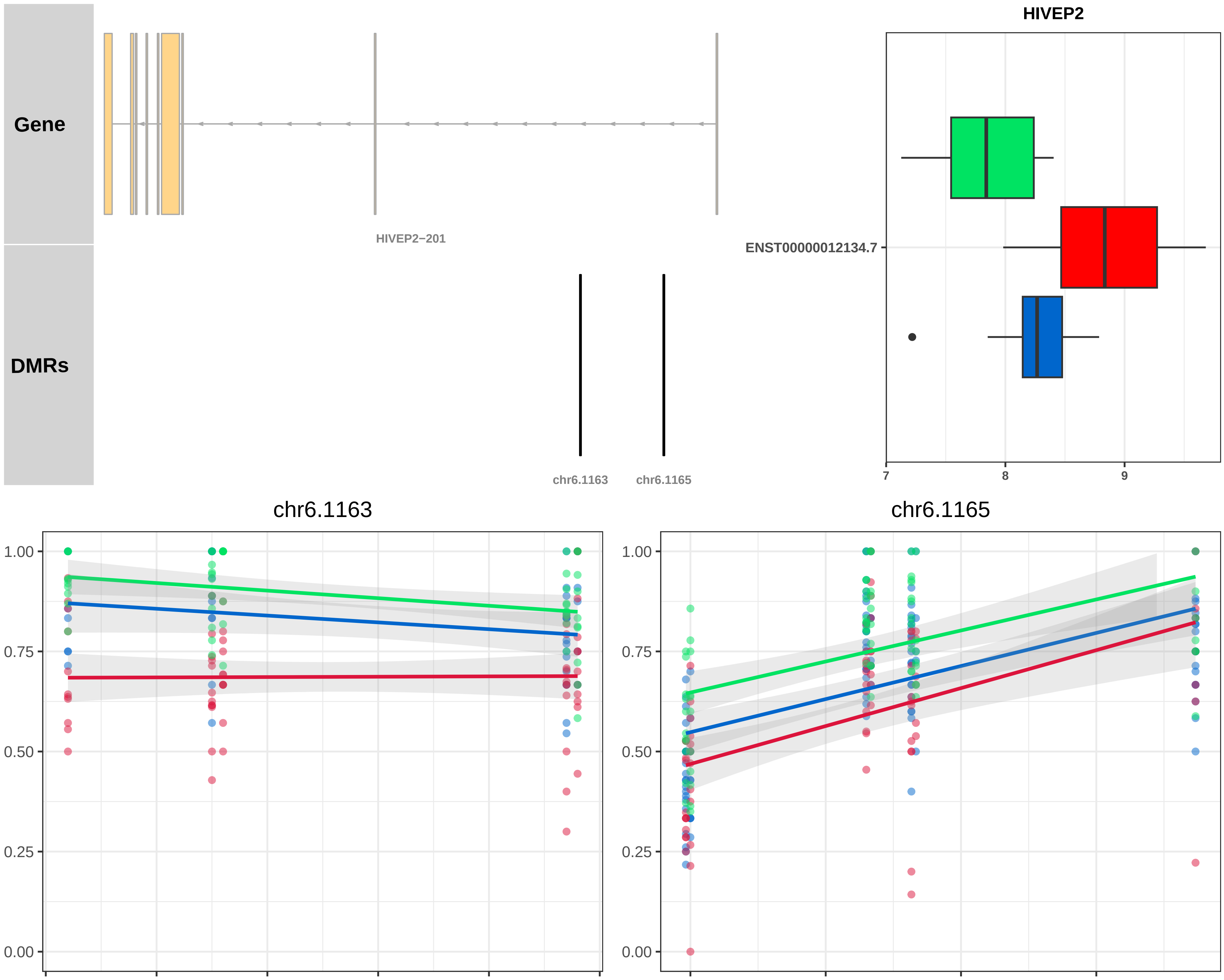

HIVEP2
Gene
DMRs
HIVEP2−201
chr6.1163
chr6.1165
ENST00000012134.7
7
8
9
chr6.1163
chr6.1165
1.00
0.75
0.50
0.25
0.00

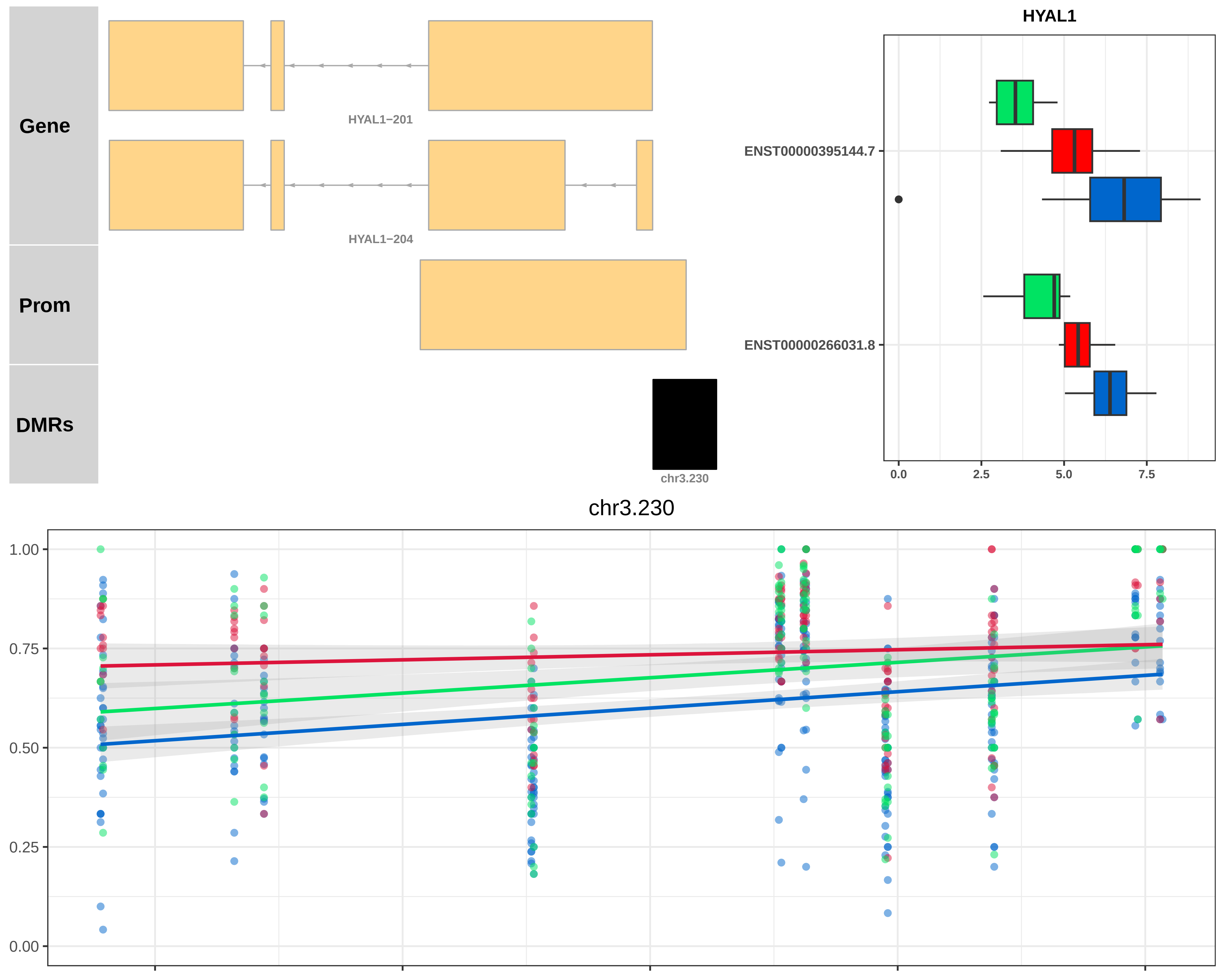

Gene
Prom
DMRs
HYAL1−201
HYAL1−204
chr3.230
HYAL1
ENST00000395144.7
ENST00000266031.8
0.0
2.5
5.0
7.5
chr3.230
1.00
0.75
0.50
0.25
0.00

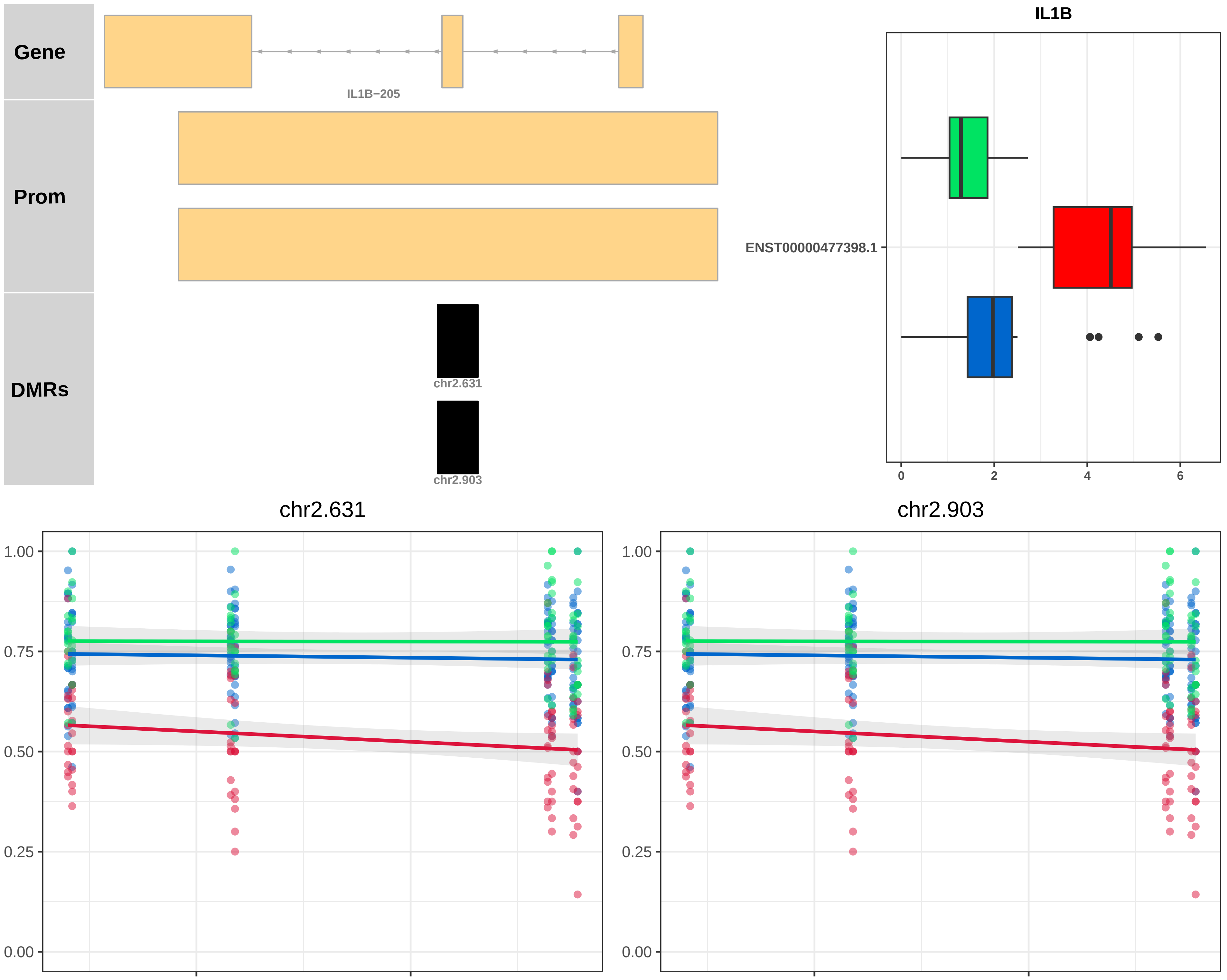

Gene
Prom
DMRs
IL1B-205
chr2.631
chr2.903
IL1B
ENST00000477398.1
0
2
4
6
chr2.631
chr2.903
1.00
0.75
0.50
0.25
0.00

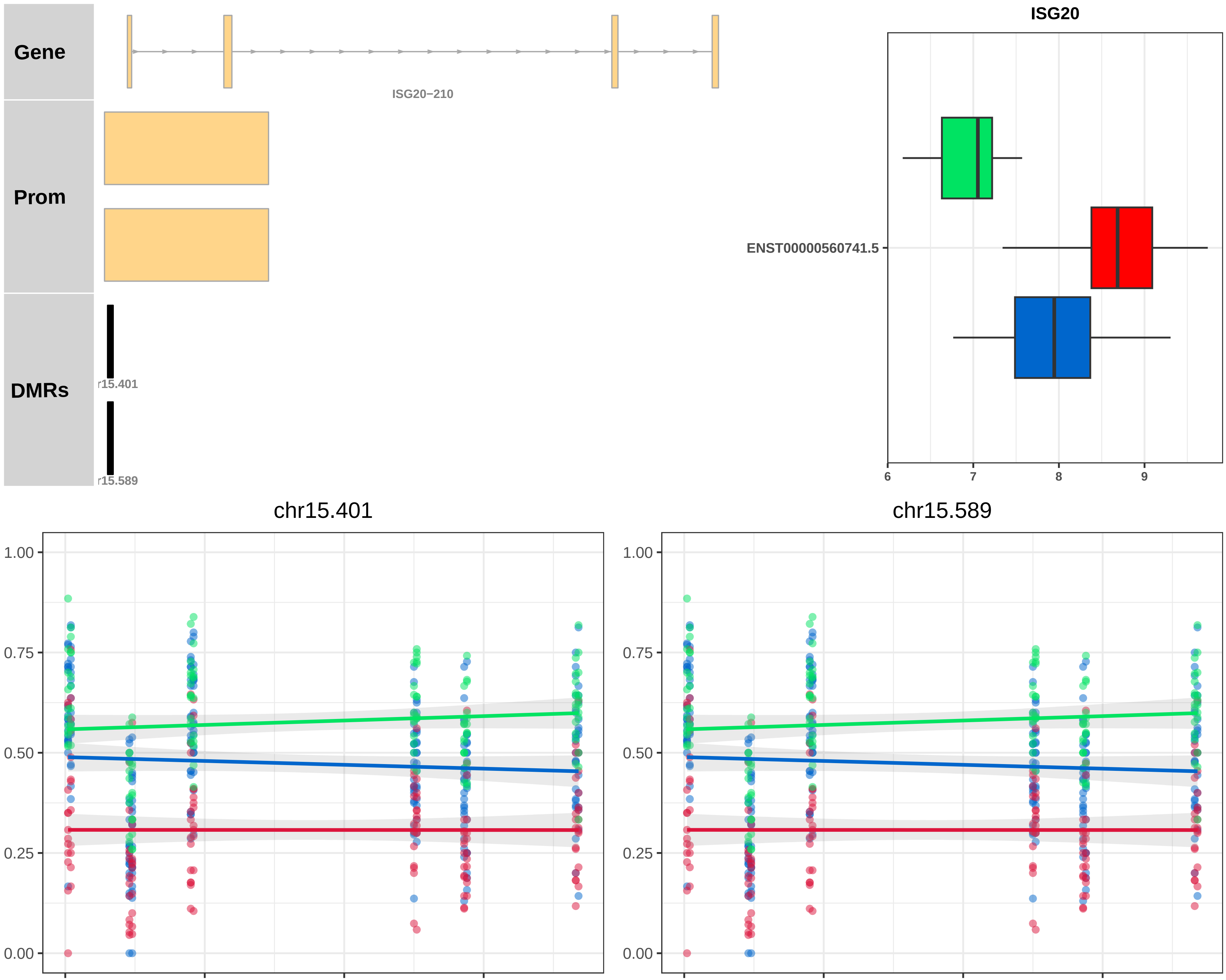

Gene
ISG20−210
Prom
DMRs
r15.401
r15.589
ISG20
ENST00000560741.5
6
7
8
9
chr15.401
chr15.589
1.00
0.75
0.50
0.25
0.00

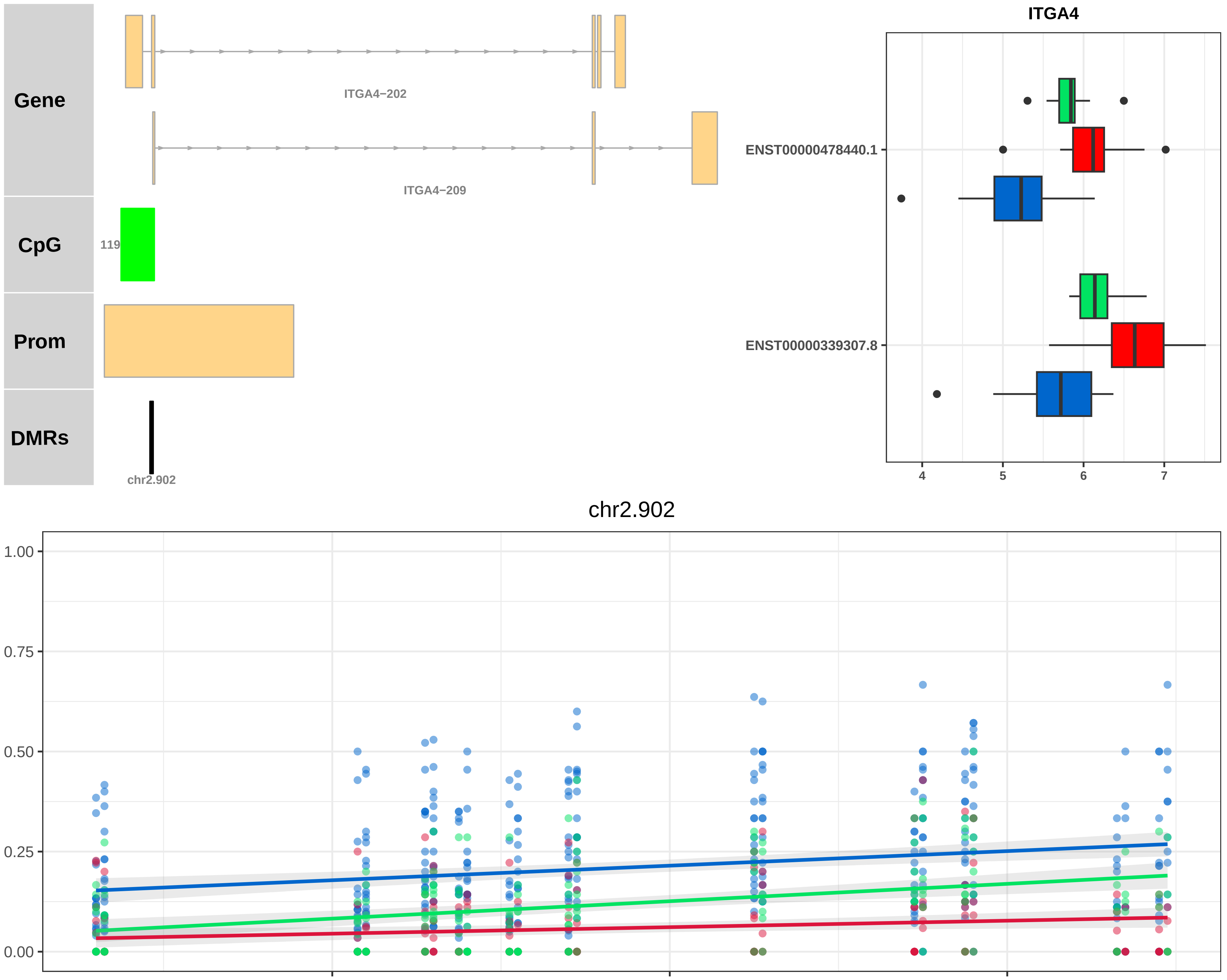

Gene
CpG
Prom
DMRs
ITGA4−202
ITGA4−209
119
chr2.902
ITGA4
ENST00000478440.1
ENST00000339307.8
4
5
6
7
chr2.902
1.00
0.75
0.50
0.25
0.00

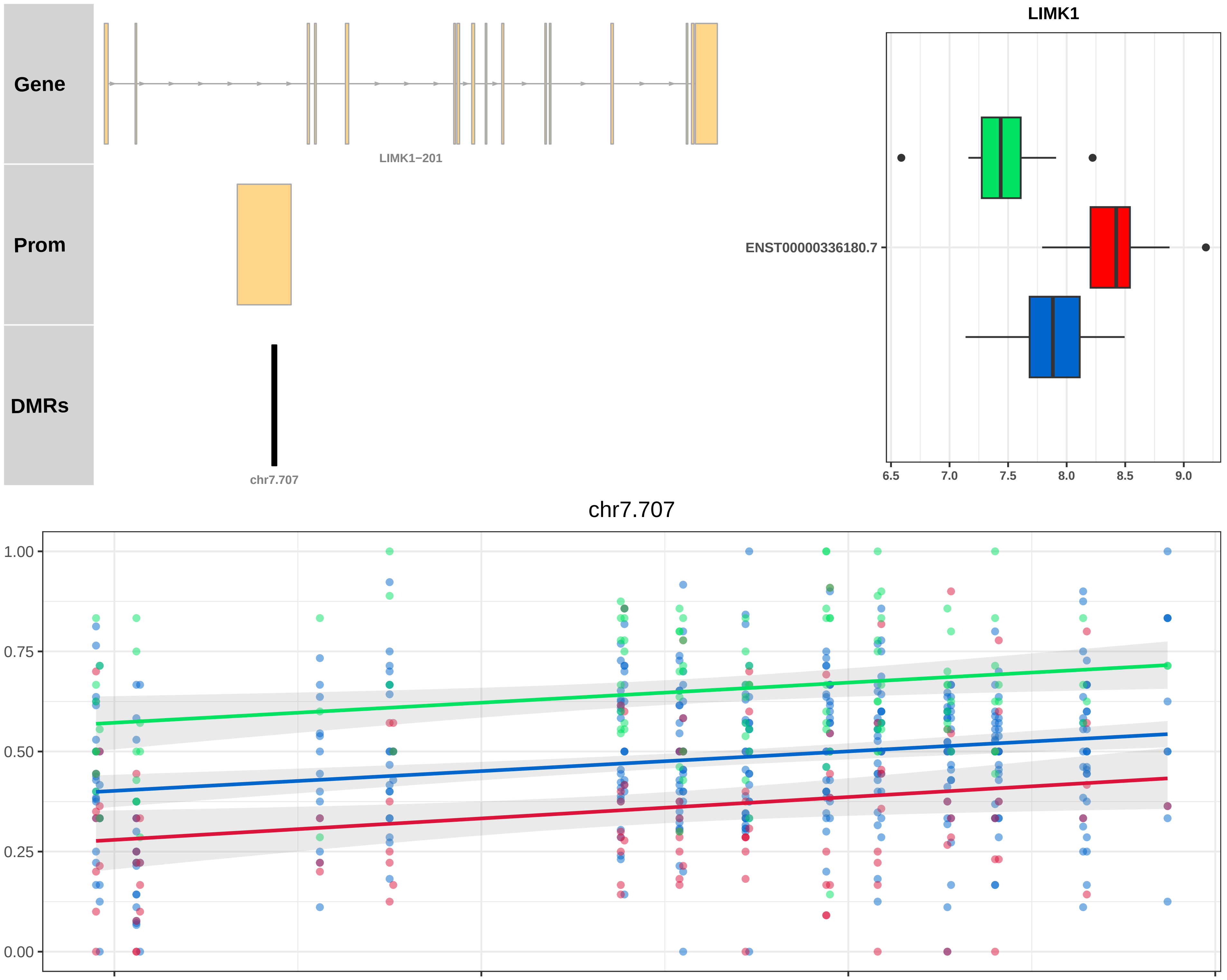
Gene
Prom
DMRs
LIMK1−201
chr7.707
LIMK1
ENST00000336180.7
6.5
7.0
7.5
8.0
8.5
9.0
chr7.707
1.00
0.75
0.50
0.25
0.00

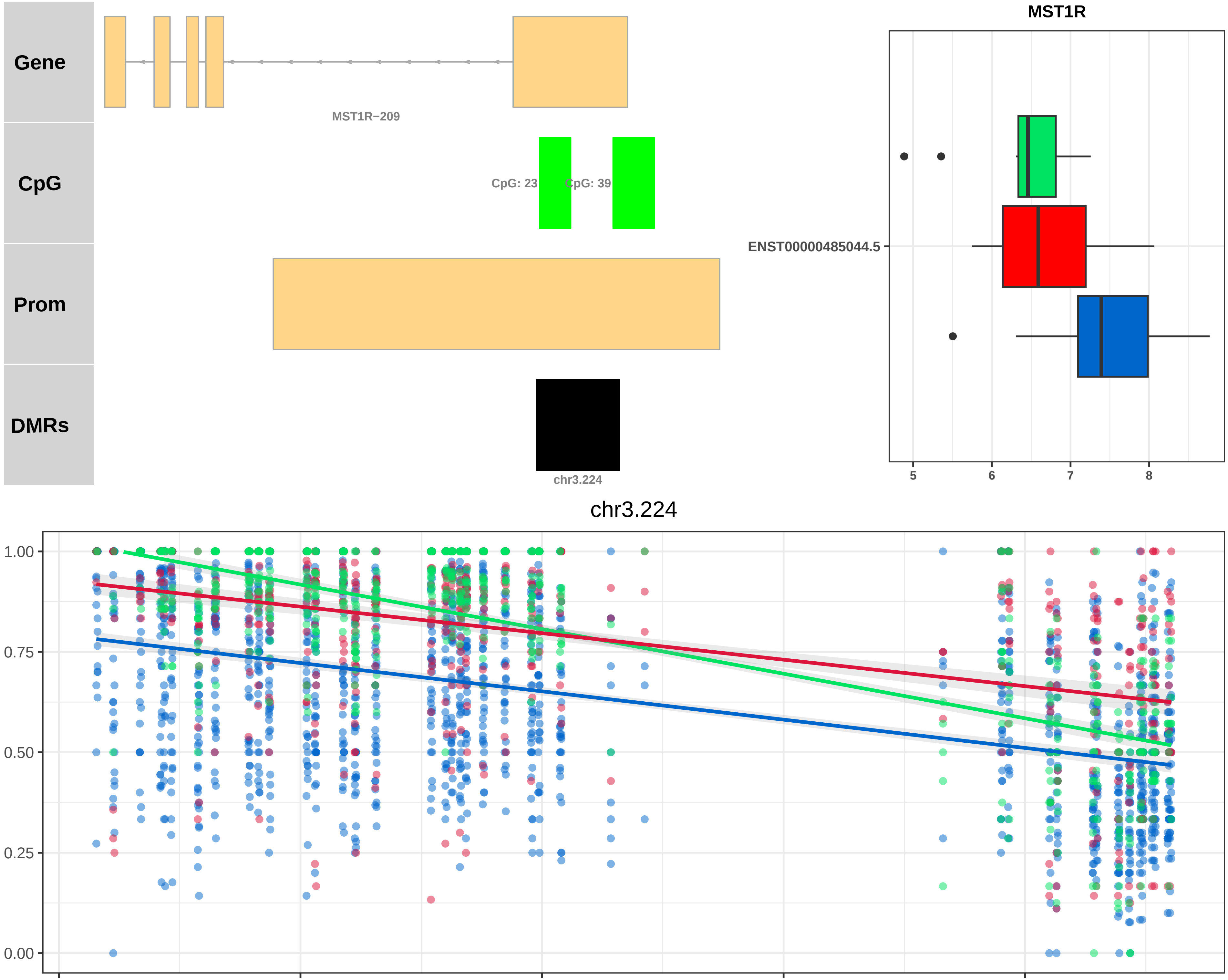

Gene
CpG
Prom
DMRs
MST1R−209
CpG: 23
CpG: 39
chr3.224
MST1R
ENST00000485044.5
5
6
7
8
chr3.224
1.00
0.75
0.50
0.25
0.00

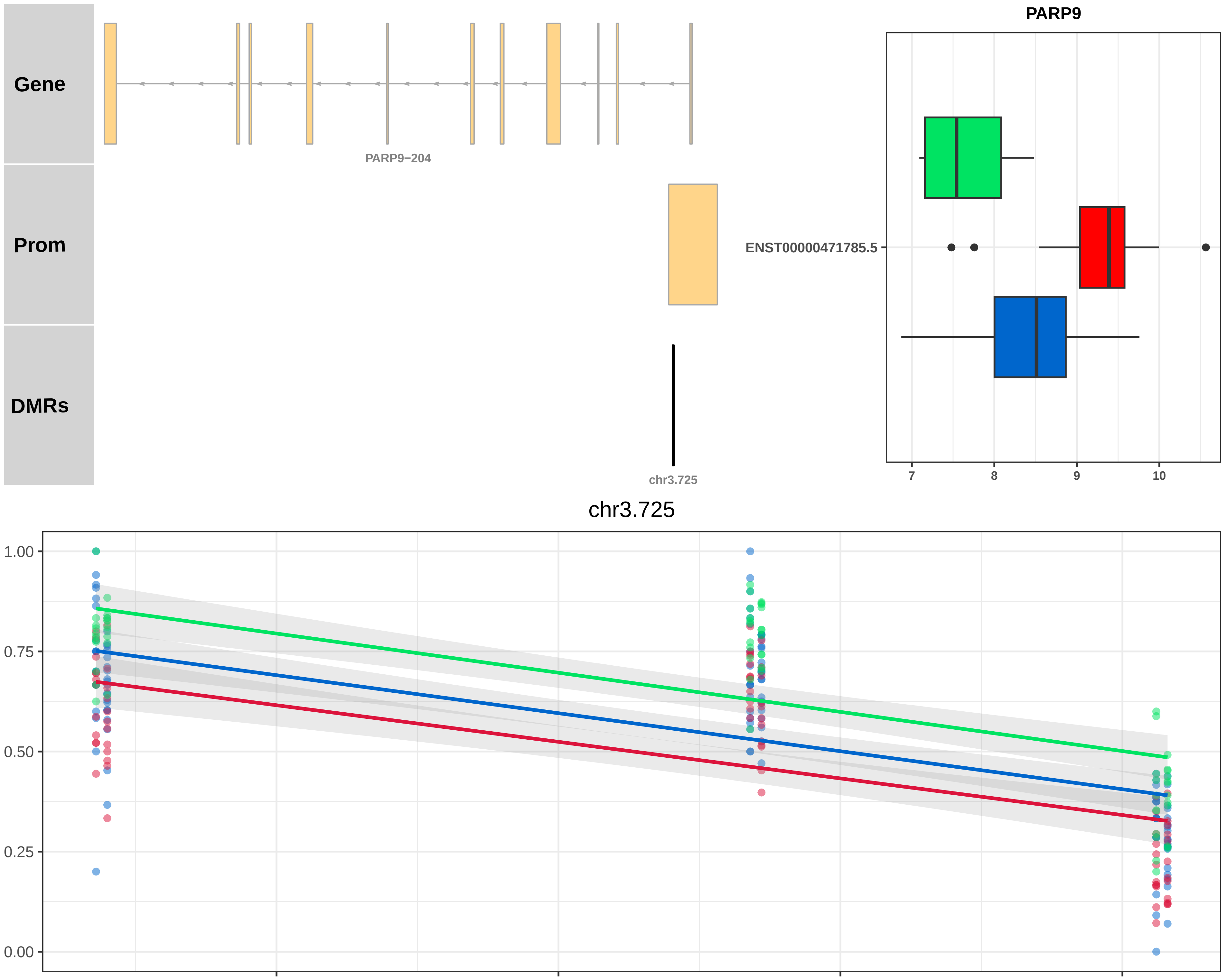

Gene
Prom
DMRs
PARP9−204
chr3.725
PARP9
ENST00000471785.5
7
8
9
10
chr3.725
1.00
0.75
0.50
0.25
0.00

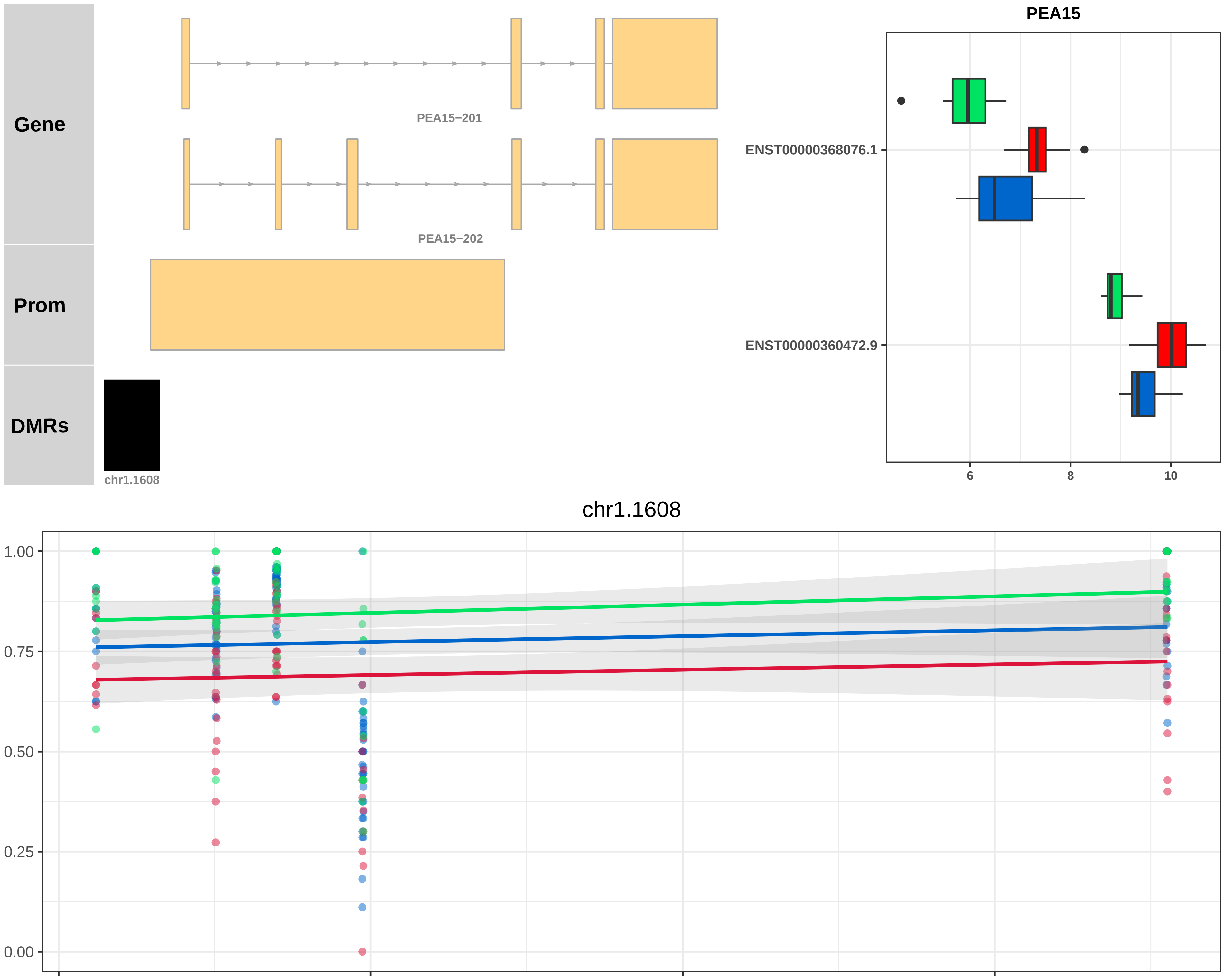
Gene
PEA15−201
PEA15−202
Prom
DMRs
chr1.1608
PEA15
ENST00000368076.1
ENST00000360472.9
6
8
10
chr1.1608
1.00
0.75
0.50
0.25
0.00

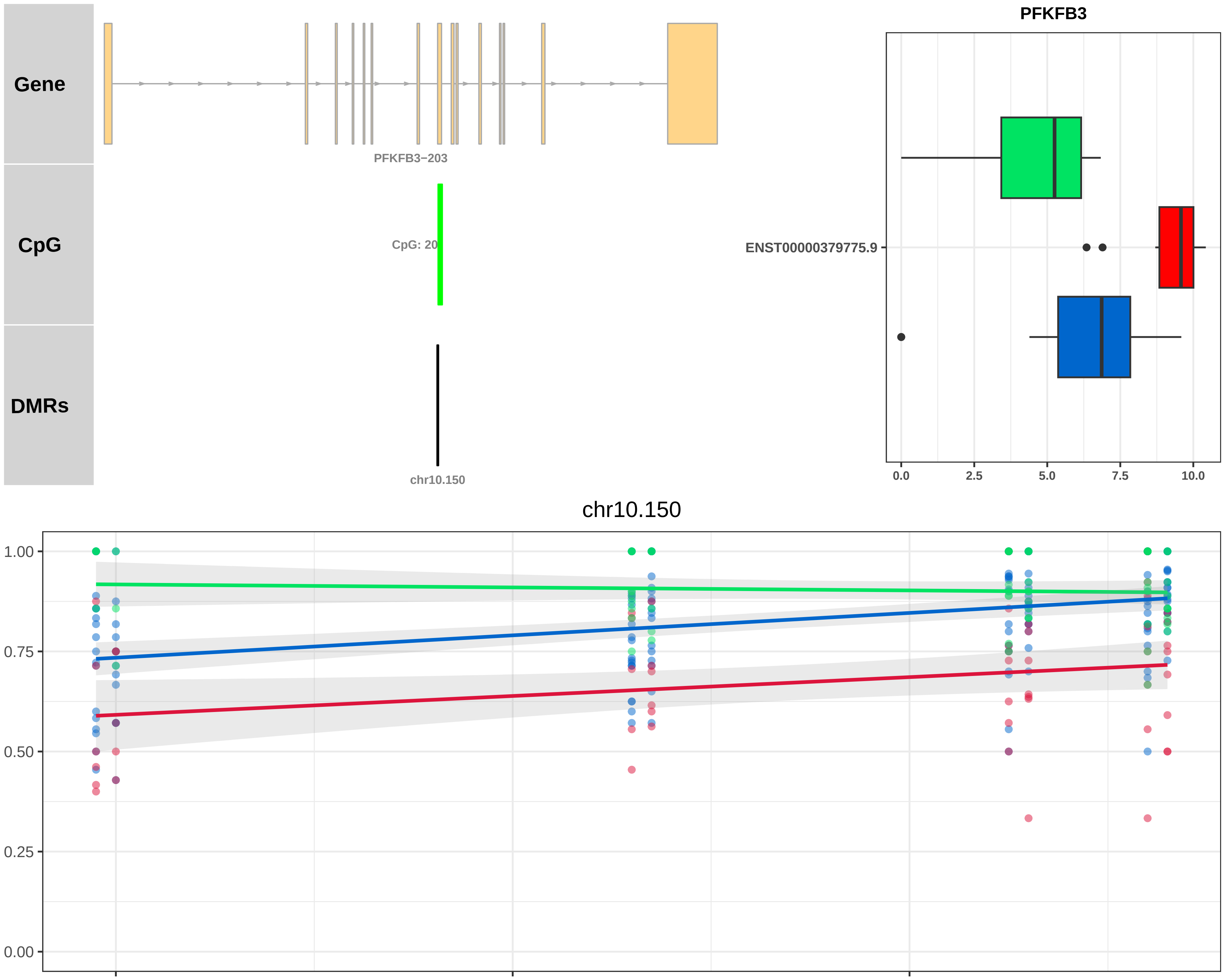

Gene
CpG
DMRs
PFKFB3−203
CpG: 20
chr10.150
PFKFB3
ENST00000379775.9
0.0
2.5
5.0
7.5
10.0
chr10.150
1.00
0.75
0.50
0.25
0.00

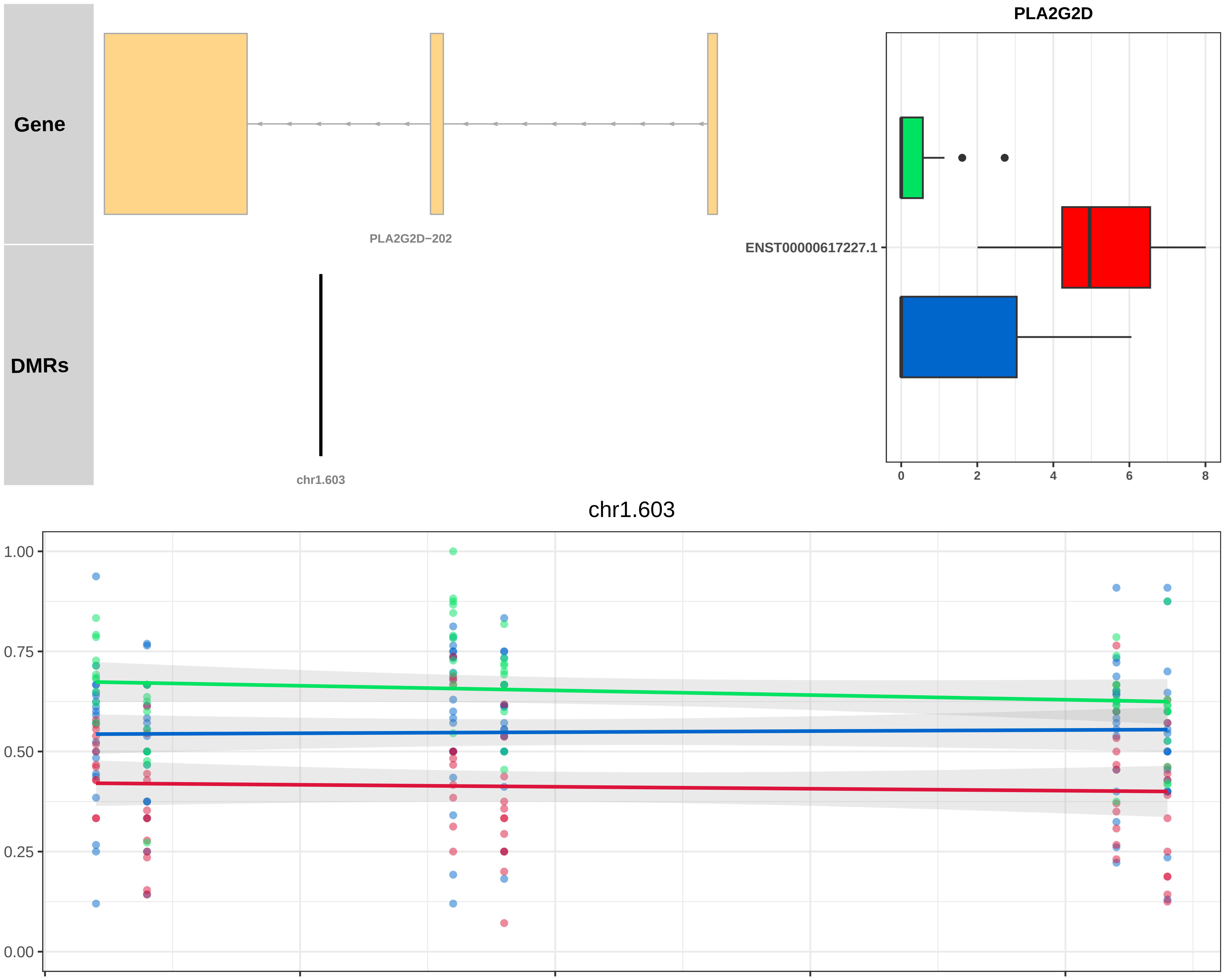

Gene
DMRs
PLA2G2D−202
chr1.603
PLA2G2D
ENST00000617227.1
0
2
4
6
8
chr1.603
1.00
0.75
0.50
0.25
0.00

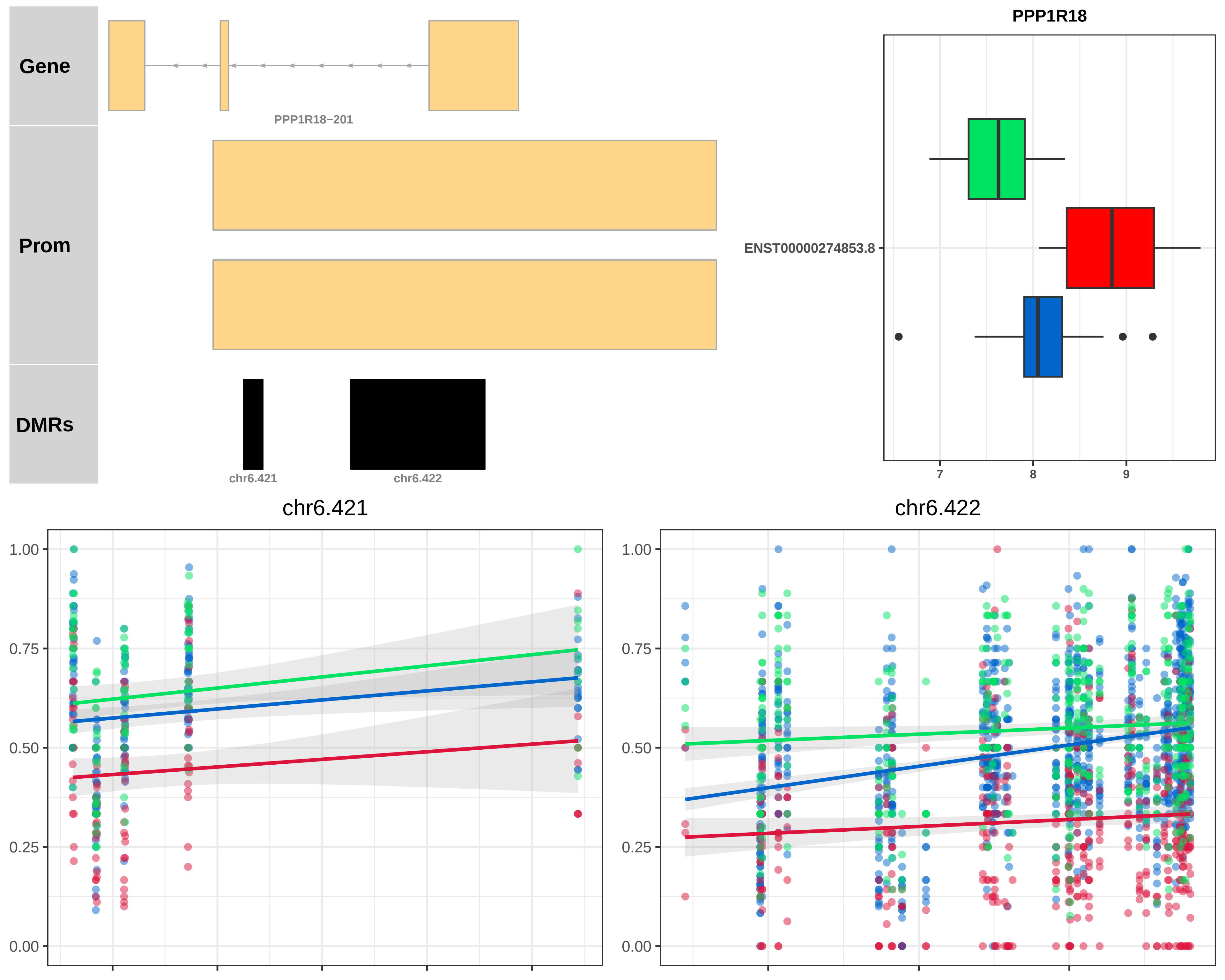
Gene
Prom
DMRs
PPP1R18−201
chr6.421
chr6.422
PPP1R18
ENST00000274853.8
7
8
9
chr6.421
chr6.422
1.00
0.75
0.50
0.25
0.00
1.00
0.75
0.50
0.25
0.00

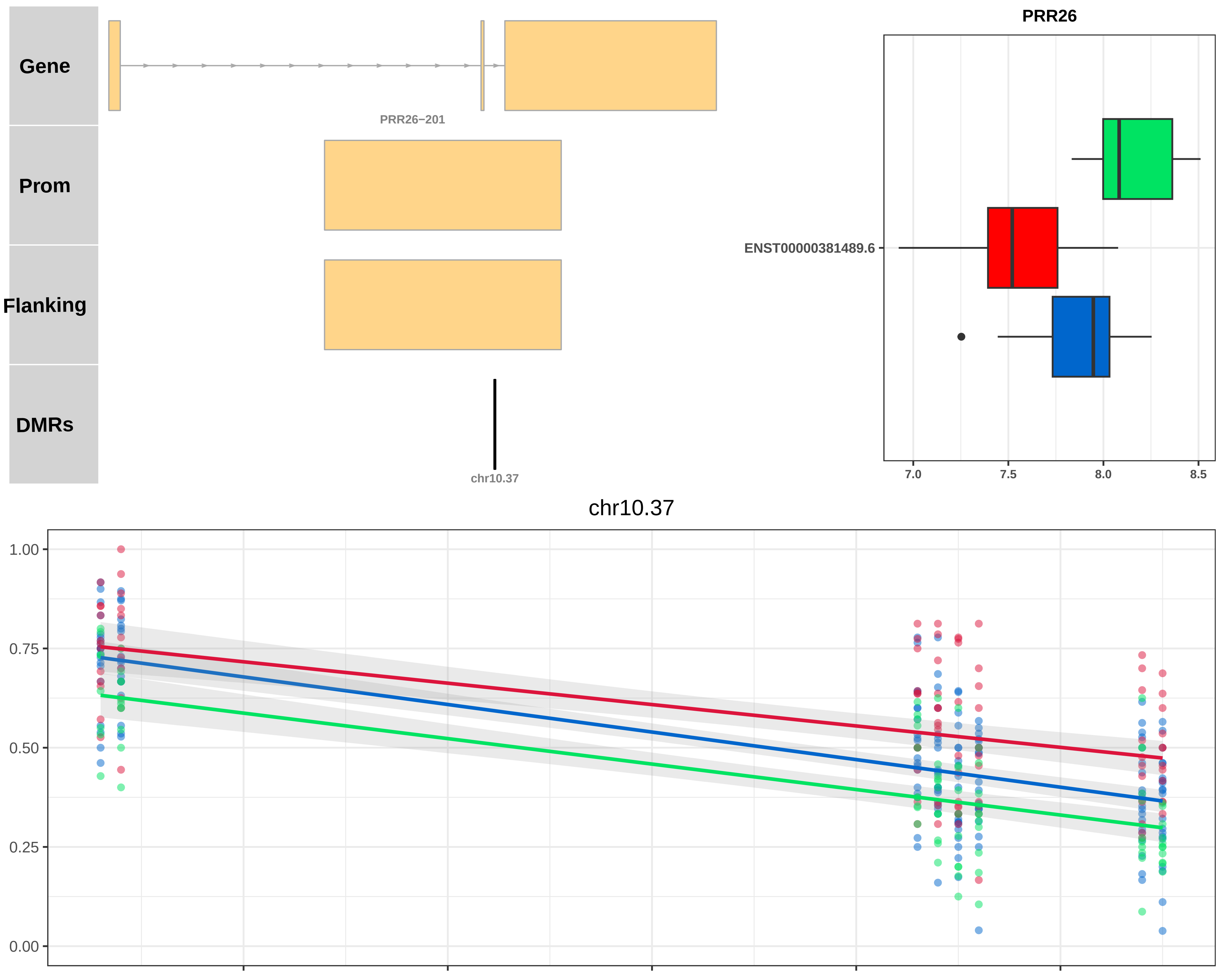

Gene
Prom
Flanking
DMRs
PRR26−201
chr10.37
PRR26
ENST00000381489.6
7.0
7.5
8.0
8.5
chr10.37
1.00
0.75
0.50
0.25
0.00

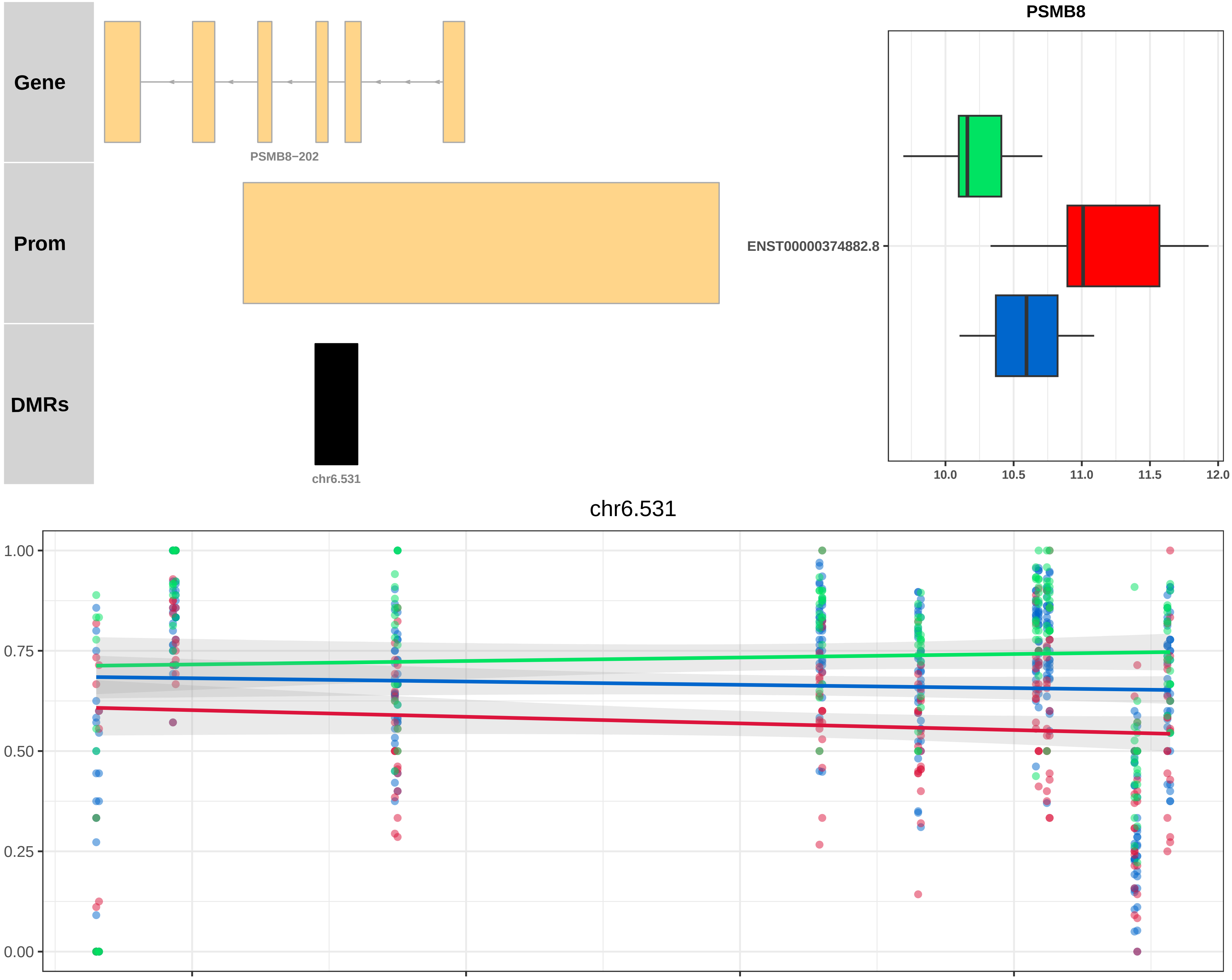

Gene
Prom
DMRs
PSMB8−202
chr6.531
PSMB8
ENST00000374882.8
10.0
10.5
11.0
11.5
12.0
chr6.531
1.00
0.75
0.50
0.25
0.00

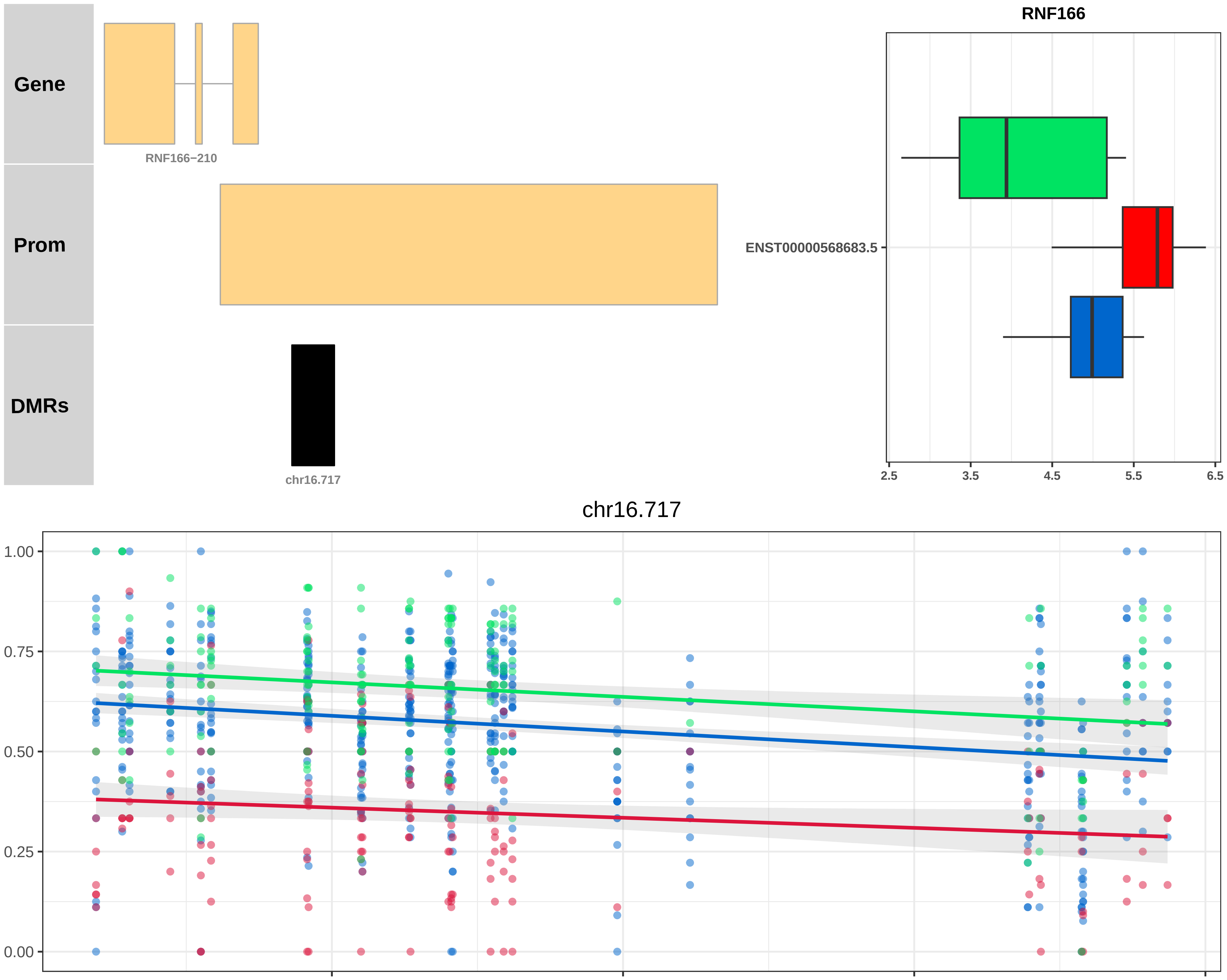
Gene
Prom
DMRs
RNF166−210
chr16.717
RNF166
ENST00000568683.5
2.5
3.5
4.5
5.5
6.5
chr16.717
1.00
0.75
0.50
0.25
0.00

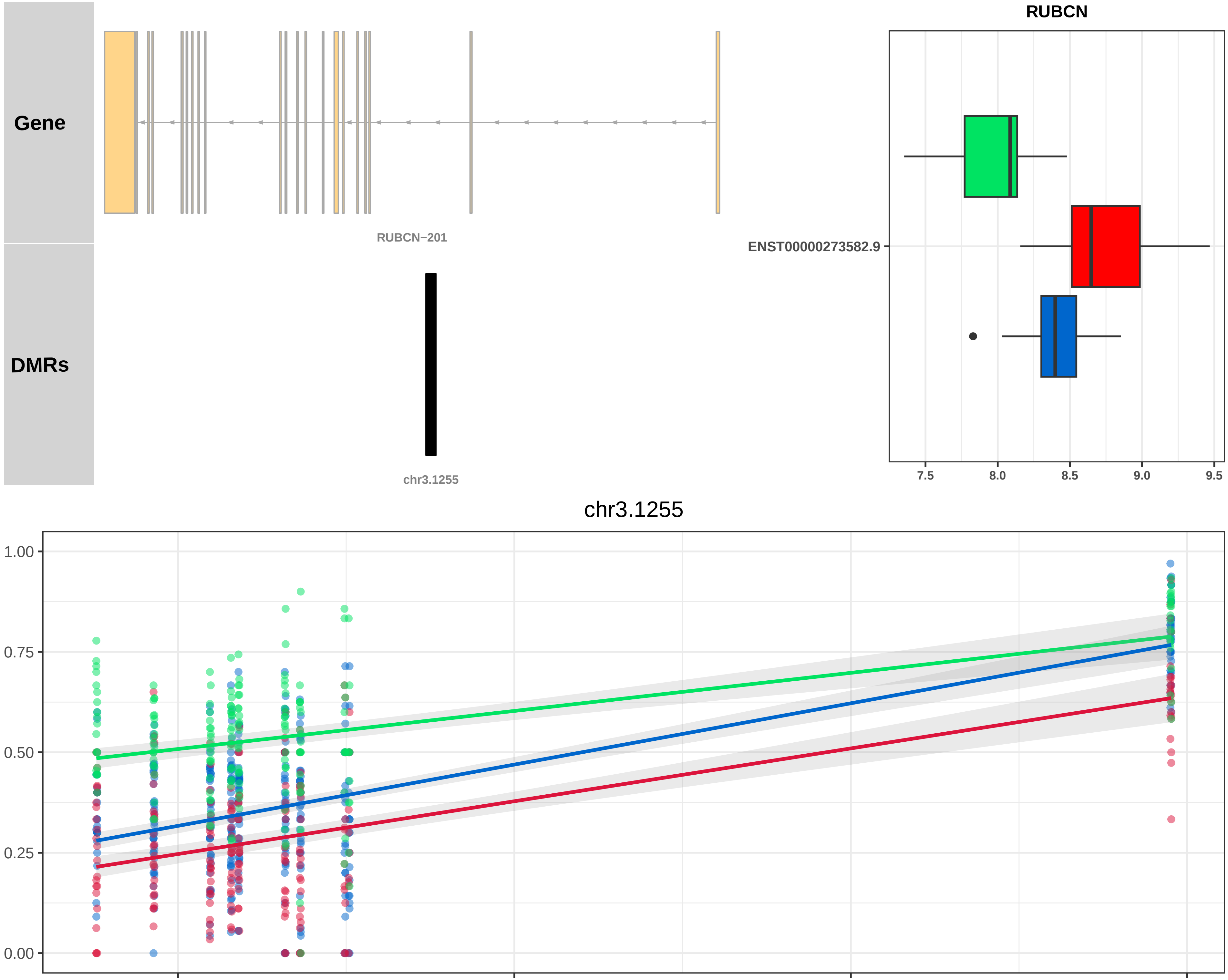
Gene
DMRs
RUBCN−201
chr3.1255
RUBCN
ENST00000273582.9
7.5
8.0
8.5
9.0
9.5
chr3.1255
1.00
0.75
0.50
0.25
0.00

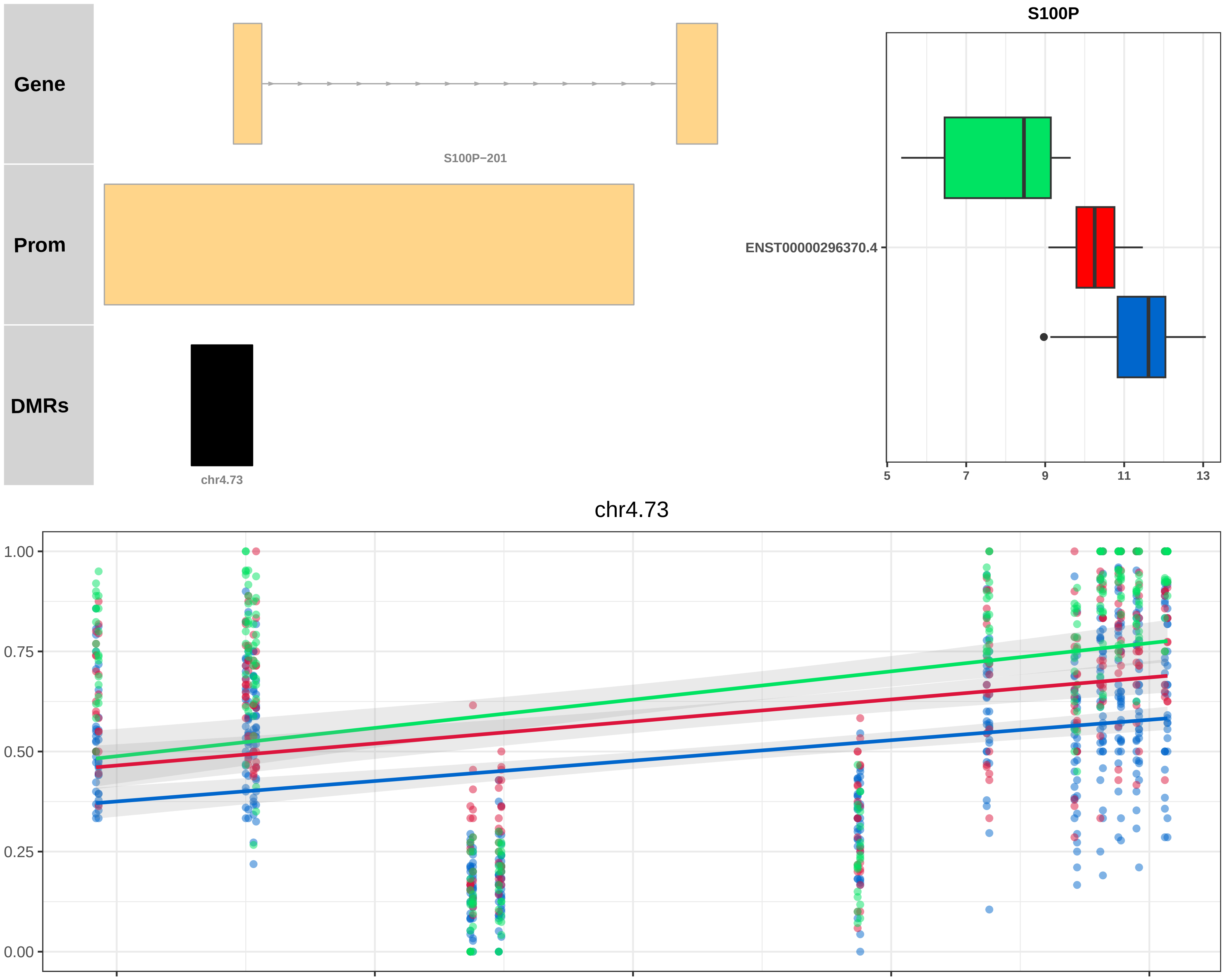

Gene
Prom
DMRs
S100P-201
chr4.73
S100P
ENST00000296370.4
5
7
9
11
13
chr4.73
1.00
0.75
0.50
0.25
0.00

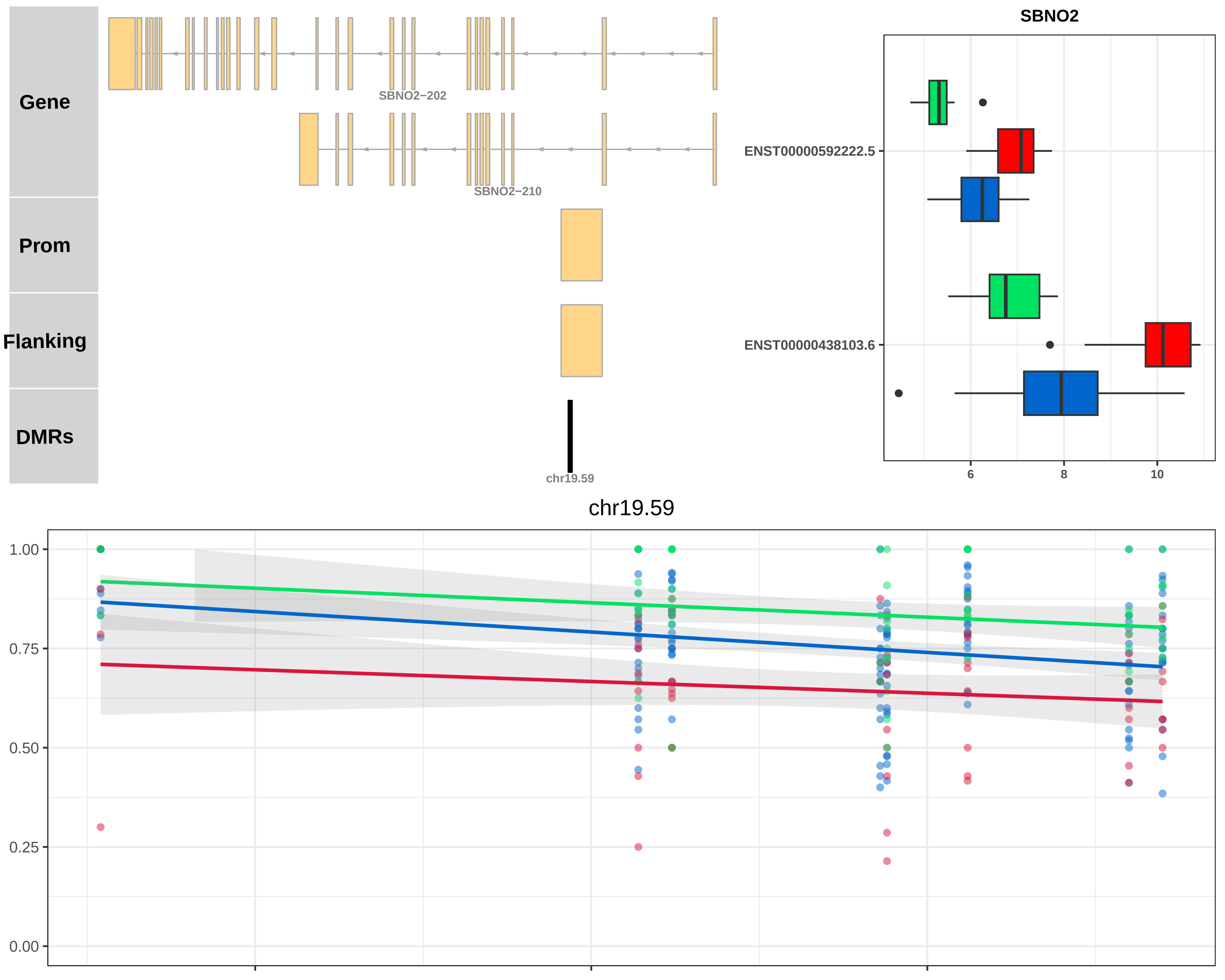

Gene
Prom
Flanking
DMRs
SBNO2−202
SBNO2−210
chr19.59
SBNO2
ENST00000592222.5
ENST00000438103.6
6
8
10
chr19.59
1.00
0.75
0.50
0.25
0.00

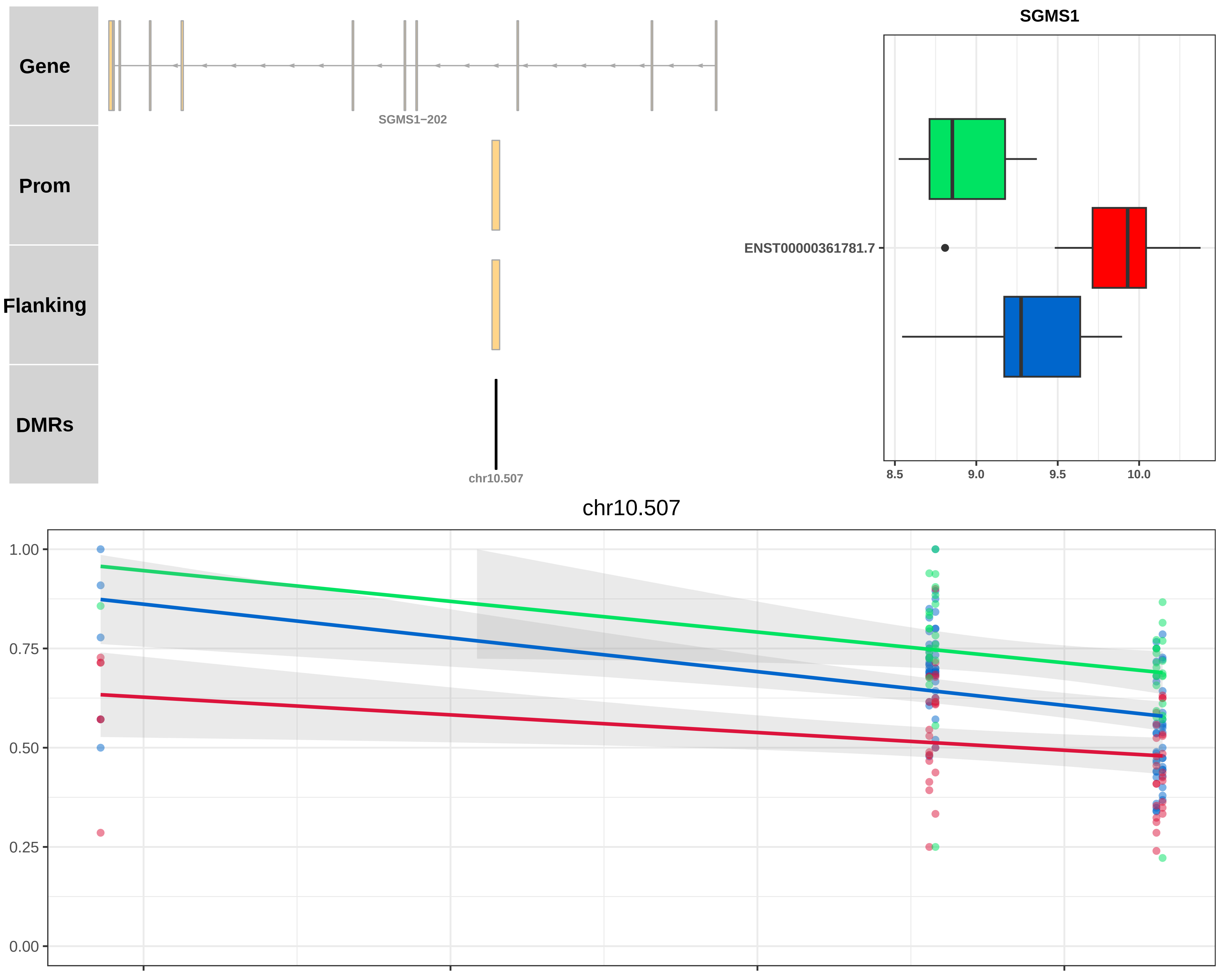

Gene
Prom
Flanking
DMRs
SGMS1−202
chr10.507
SGMS1
ENST00000361781.7
8.5
9.0
9.5
10.0
chr10.507
1.00
0.75
0.50
0.25
0.00

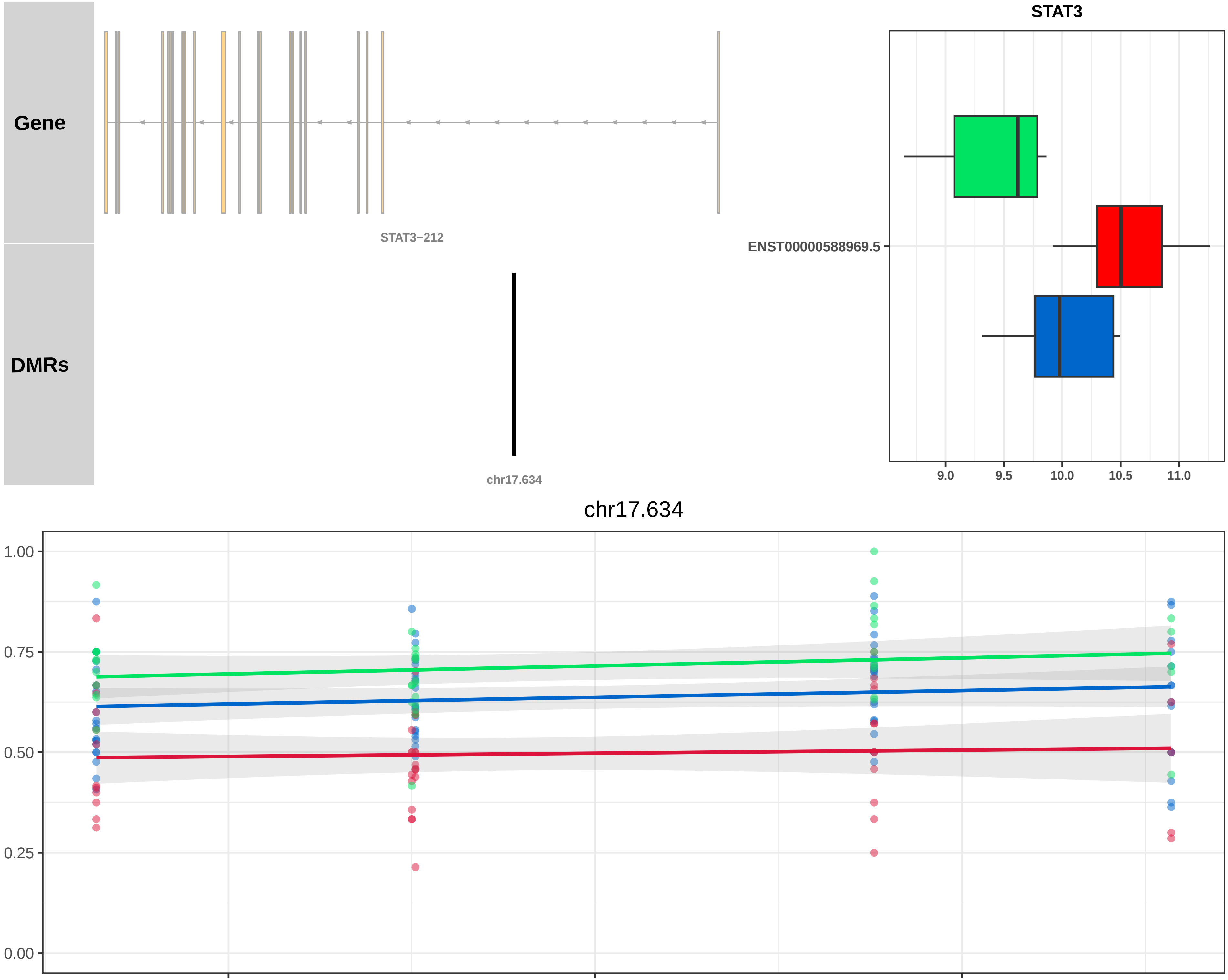
STAT3
Gene
DMRs
STAT3−212
chr17.634
ENST00000588969.5
9.0
9.5
10.0
10.5
11.0
chr17.634
1.00
0.75
0.50
0.25
0.00

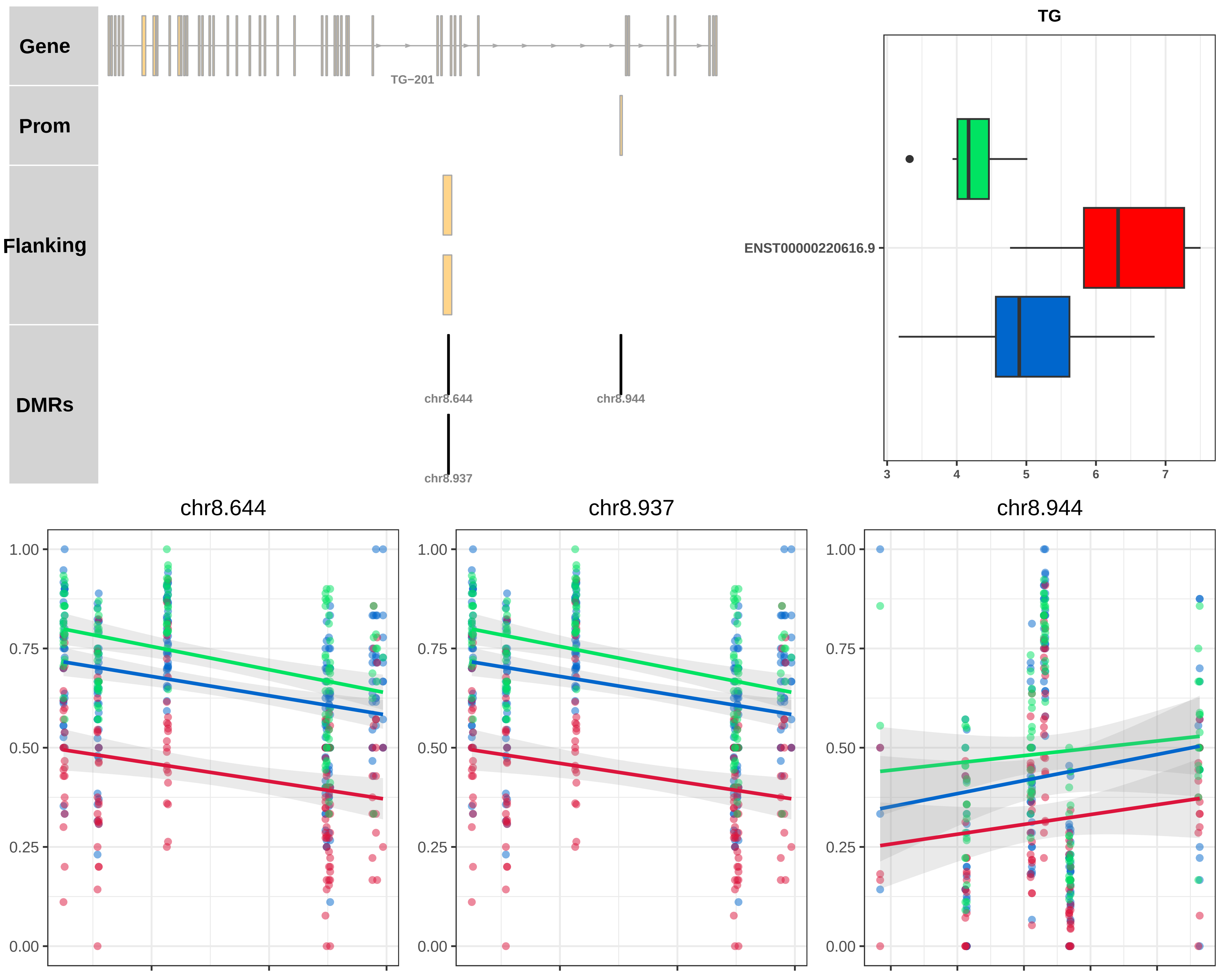

Gene
Prom
Flanking
DMRs
TG−201
chr8.644
chr8.944
chr8.937
TG
ENST00000220616.9
3
4
5
6
7
chr8.644
chr8.937
chr8.944
1.00
0.75
0.50
0.25
0.00

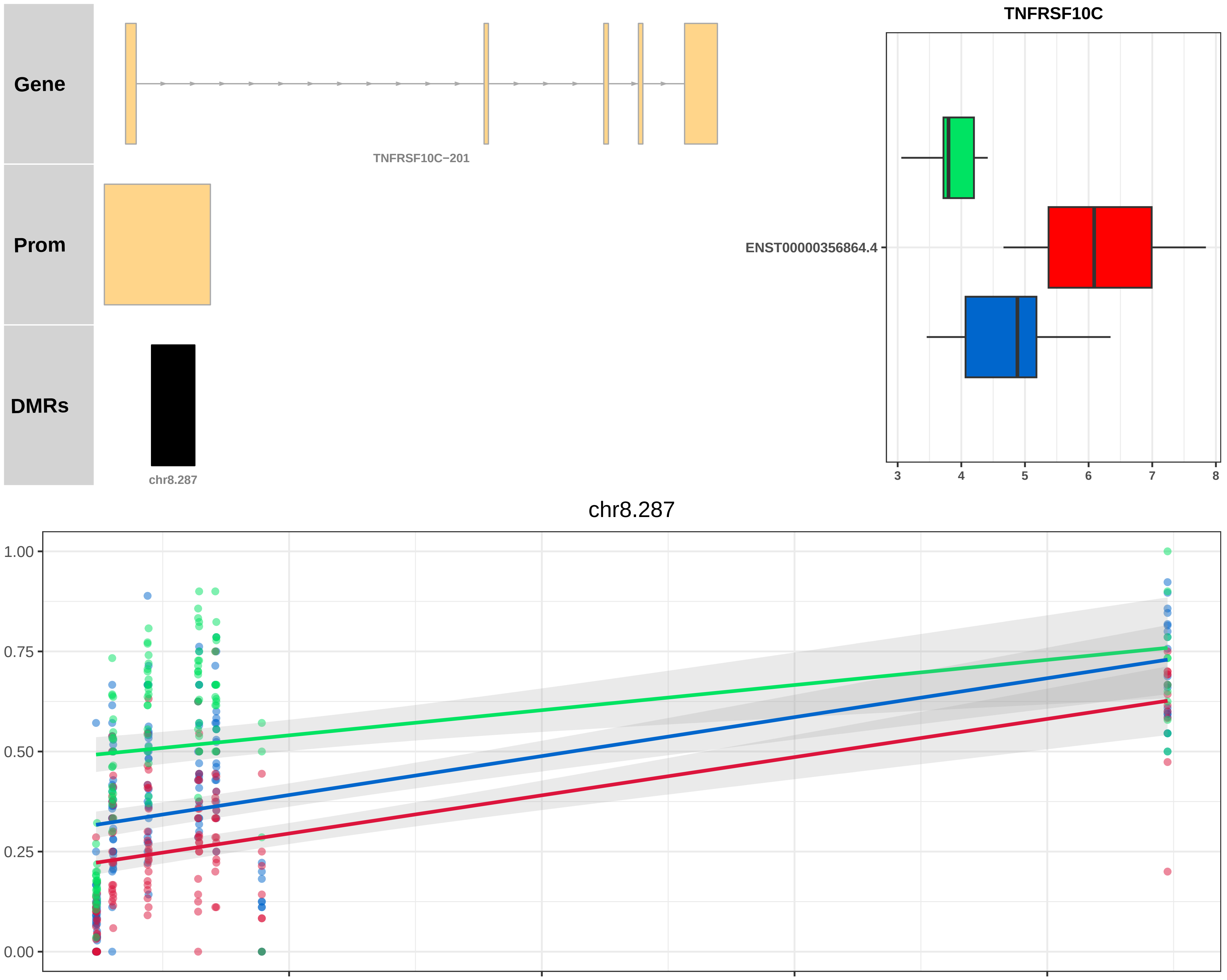
Gene
Prom
DMRs
TNFRSF10C−201
chr8.287
TNFRSF10C
ENST00000356864.4
3
4
5
6
7
8
chr8.287
1.00
0.75
0.50
0.25
0.00

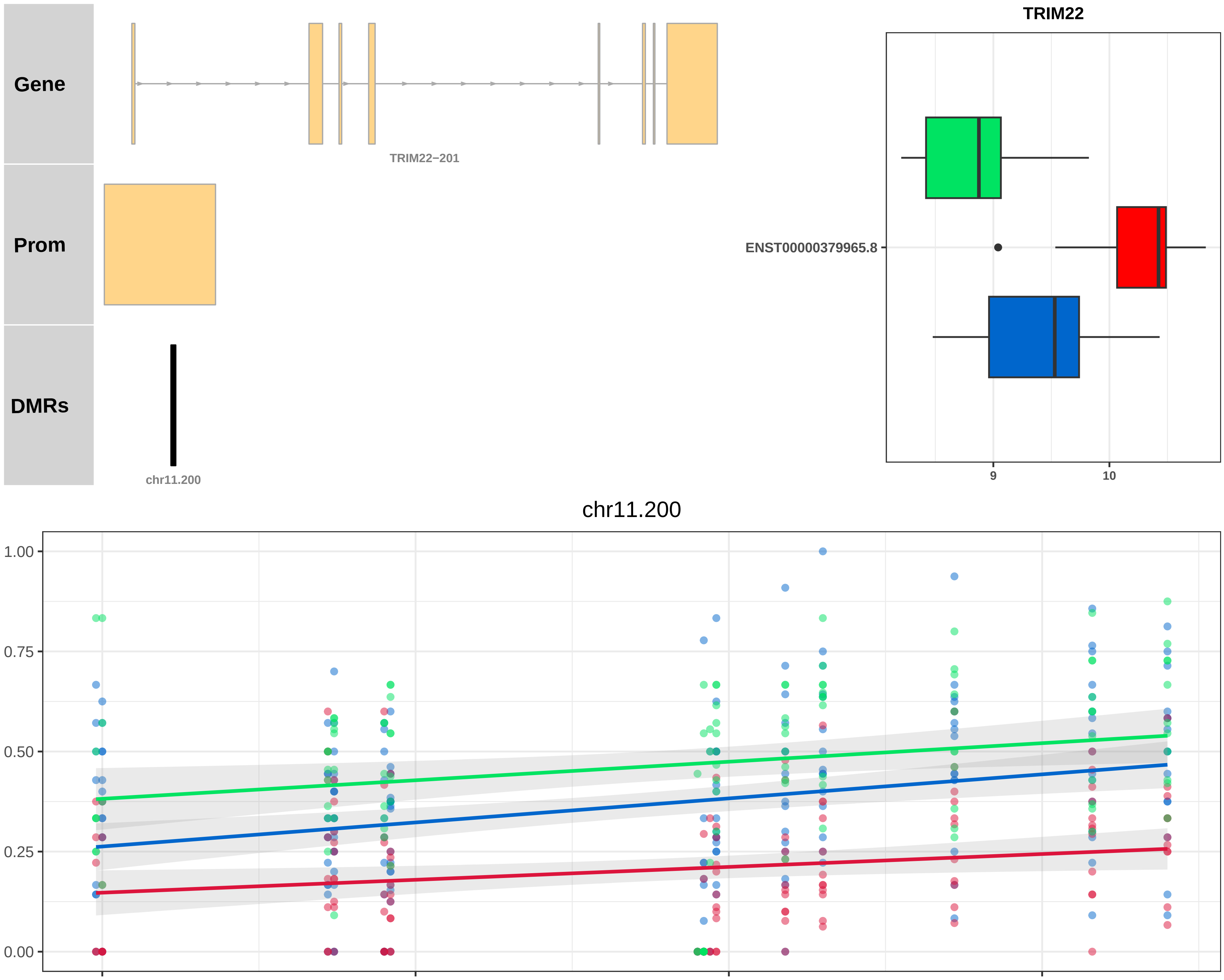

Gene
Prom
DMRs
TRIM22−201
chr11.200
TRIM22
ENST00000379965.8
9
10
chr11.200
1.00
0.75
0.50
0.25
0.00

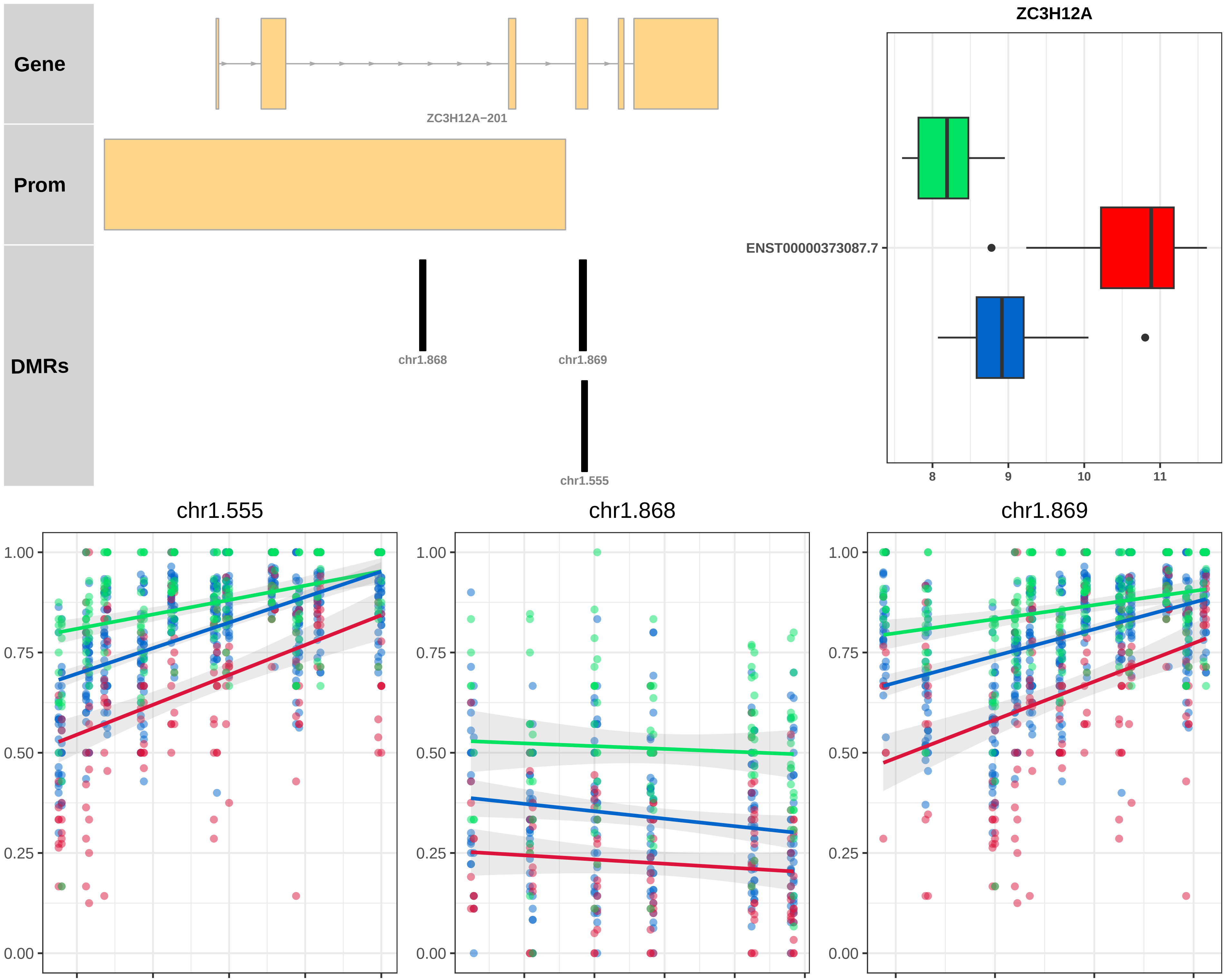
Gene
Prom
DMRs
ZC3H12A−201
chr1.868
chr1.869
chr1.555
ZC3H12A
ENST00000373087.7
8
9
10
11
chr1.555
chr1.868
chr1.869
1.00
0.75
0.50
0.25
0.00

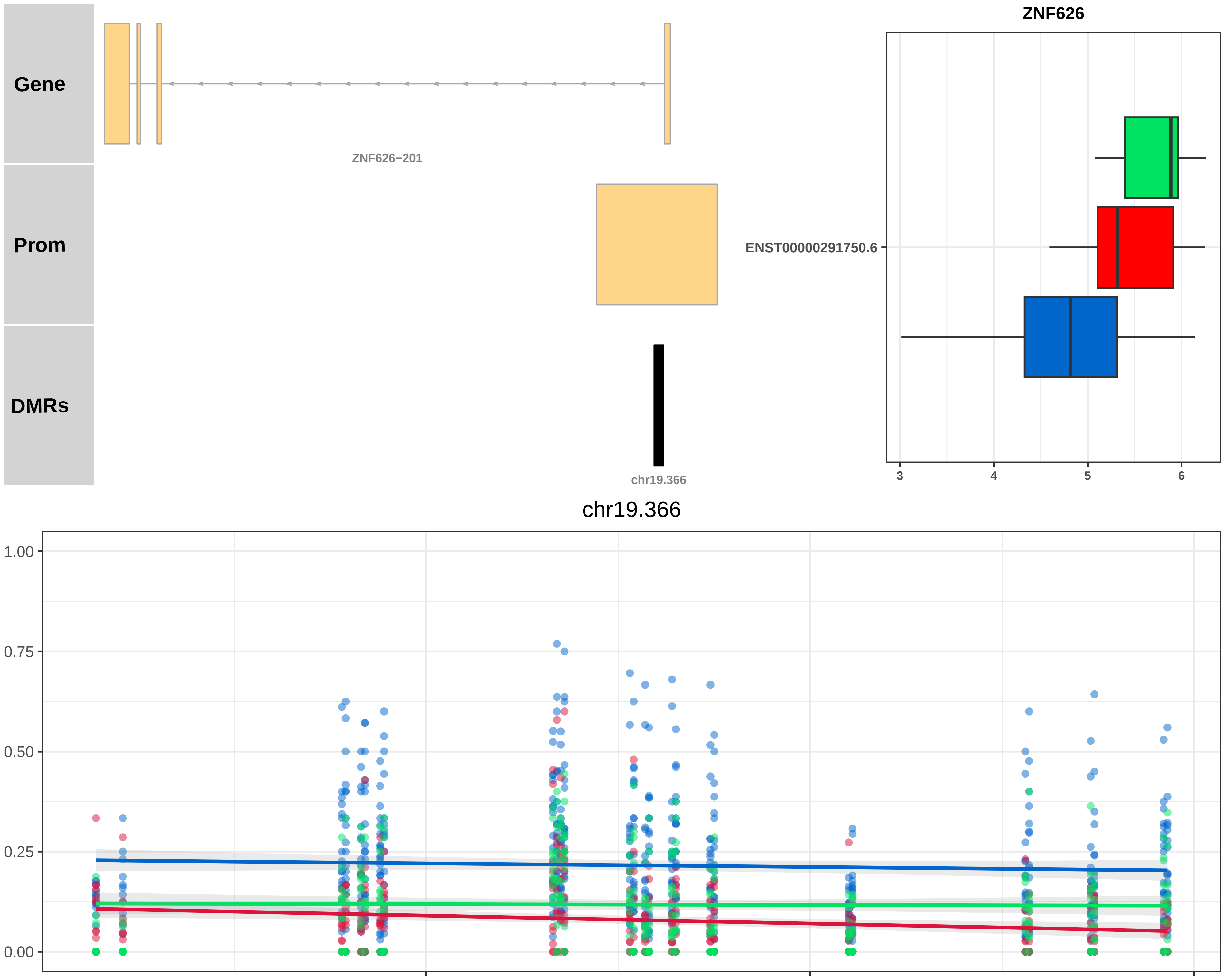

Gene
Prom
DMRs
ZNF626−201
chr19.366
ZNF626
ENST00000291750.6
3
4
5
6
chr19.366
1.00
0.75
0.50
0.25
0.00
