# Supplementary material for: DNA methylation fine-tunes pro-and anti-inflammatory signalling pathways in inactive ulcerative colitis tissue biopsies
Source: Sci Rep. 2024 Mar 21;14:6789. doi: 10.1038/s41598-024-57440-0 (PMC10957912; doi:10.1038/s41598-024-57440-0)

Supplementary data 5 shows multiple boxplots. The title of each plot indicates the cell type analyzed.
The Y axis represents the average estimated cell fraction for all control (NN), inactive UC(RM), active UC(UC) samples as indicated on the X axis

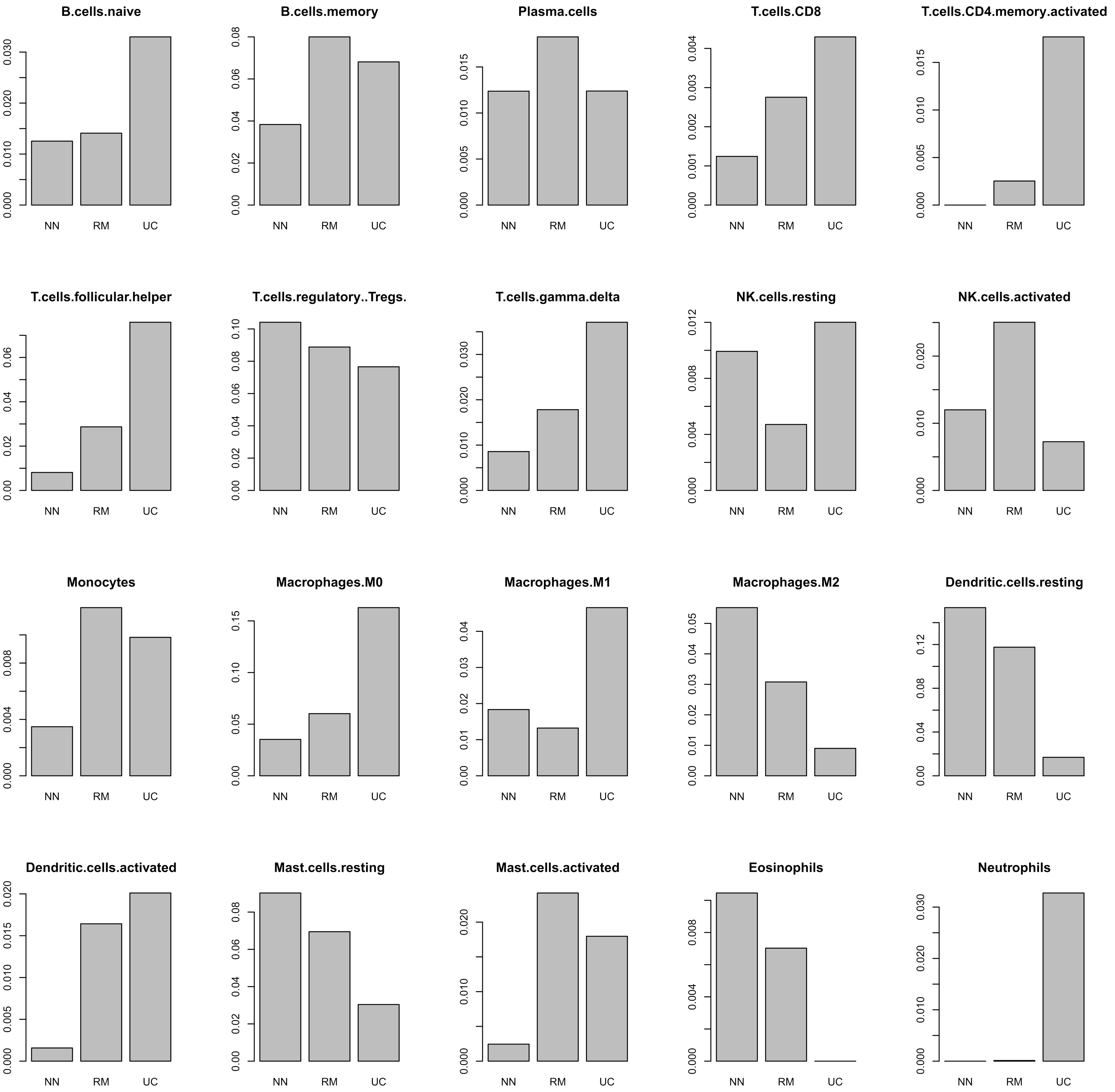

Supplement: Supplementary file 5 — Supplementary Information 5. [file 41598_2024_57440_MOESM5_ESM.pdf]
